# Data structure *set-trie* for storing and querying sets: Theoretical and empirical analysis

**DOI:** 10.1371/journal.pone.0245122

**Published:** 2021-02-10

**Authors:** Iztok Savnik, Mikita Akulich, Matjaž Krnc, Riste Škrekovski

**Affiliations:** 1 Faculty of Mathematics, Natural Sciences and Information Technologies, University of Primorska, Koper, Slovenia; 2 Faculty of Information Studies, Novo Mesto, Slovenia; 3 Faculty of Mathematics and Physics, University of Ljubljana, Ljubljana, Slovenia; Sejong University, REPUBLIC OF KOREA

## Abstract

Set containment operations form an important tool in various fields such as information retrieval, AI systems, object-relational databases, and Internet applications. In the paper, a *set-trie* data structure for storing sets is considered, along with the efficient algorithms for the corresponding set containment operations. We present the mathematical and empirical study of the set-trie. In the mathematical study, the relevant upper-bounds on the efficiency of its expected performance are established by utilizing a natural probabilistic model. In the empirical study, we give insight into how different distributions of input data impact the efficiency of set-trie. Using the correct parameters for those randomly generated datasets, we expose the key sources of the input sensitivity of set-trie. Finally, the empirical comparison of set-trie with the inverted index is based on the real-world datasets containing sets of low cardinality. The comparison shows that the running time of set-trie consistently outperforms the inverted index by orders of magnitude.

## 1 Introduction

Given a set of sets *S* and a set *X* of symbols from some alphabet Σ, a set containment query searches either for the subsets of *X* from *S*, or, the supersets of *X* from *S*. There are two *existence* set containment queries. The query existsSubset returns true if there exists some *Y* ∈ *S* such that *Y* ⊆ *X*, or false otherwise. Similarly, the query existsSuperset returns true if a superset of *X* exists in *S*. The *retrieval* set containment queries are also two. The query getAllSubsets returns all subsets of *X* from *S*, and the query getAllSupersets returns all supersets of *X* from *S*.

The problem that we address in this paper is the design of an index data structure that provides efficient set containment operations. Let us first present the motivation for the design of the index data structure.

### 1.1 The motivation

The efficient data structure for querying sets of sets and, in particular, for answering the set containment queries is needed in several software systems, including the Web search engines, data mining tools, object-relational databases, rule-based expert systems, AI planning systems, and Internet applications. In this section we give some insight into the use of set containment operations in these areas.

In *information retrieval* (abbr. IR), the text files are represented using a bag-of-words model [1]. In other words, the text is treated as a multiset of words where the words in IR have the role of an alphabet. The most basic operation in an IR system inquires about the documents that contain a given word. A multiset containment query is a conjunctive boolean query where the keywords that constitute a query are connected with logical conjunction. The query searches for documents (multisets of words) that contain all keywords from a query. Traditionally, IR systems use the inverted index for querying a collection of texts [2]. However, there are proposals for using other indexes. For instance, a signature tree S-tree [3] is also used for indexing texts [4, 5].

The enumeration of subsets of a given universal set Σ is very common in *data mining* algorithms [6], where sets are used as the basis for the representation of hypotheses, and the search space forms a lattice. Often, we have to know if the algorithm has already evaluated a given hypothesis. This can be checked by searching the set of hypotheses (sets) that have already been processed. Furthermore, in some cases, the hypotheses can be easily overthrown if a superset hypothesis has already been shown not valid. Such problems include the discovery of association rules and functional dependencies as well as some forms of propositional logic [7–9].

Sets form the framework for the implementation of systems from some other AI areas too. For example, *rule-based expert systems* use the set containment queries to implement fast pattern-matching algorithms that determine which rules are fired in each cycle of the expert system execution. Here the sets form pre-conditions of rules composed of the elementary conditions. Given a set of valid conditions, the set of fired rules includes those with the pre-condition included in this set [10, 11]. Further, in *AI planning systems*, the goal sets are used to store the goals to be achieved from a given initial state. Planning modules use the subset queries in the procedure that examines if a given goal set is satisfiable. A part of the procedure represents querying the goal sets that were previously shown to be unsatisfiable. Here also the sets are used to form the basic structure of hypothesis space [12].

*Object-relational database management systems*, use tables with set-valued attributes, i.e., the attributes that range over sets. The set containment queries can express either the selection or join operation based on the set containment condition. Efficient access to the relational records based on the conditions that involve set operations are vital for the fast implementation of such queries [5, 13, 14]. Further, the set containment join is a problem that received significant research interest in the last two decades [13, 15–19].

Finally, querying sets of sets is becoming essential in *Internet applications*. The objects in the Internet applications are often represented by a set of features. Two operations, in particular, recently attracted much attention. Firstly, given the collection of objects and a query object, the similarity search retrieves all objects similar to a query object [20–22]. Secondly, given two sets of objects, the exact set similarity join finds all pairs of similar objects [23–25]. The operations are used for data cleaning, information integration, entity detection, near duplicate detection and personalized recomendation.

### 1.2 Proposed in-memory index data structure

We propose a novel in-memory index data structure *set-trie* that efficiently implements the set containment queries. The initial implementation of the set-trie has been done in the frame of the data-mining system fdep [26]. The preliminary study of set-trie based on the multisets has been published in [27]. In this paper, we present the theoretical analysis of set-trie and the comprehensive empirical analysis of set-trie, including the comparison of set-trie with the inverted index. The empirical analysis was performed on the artificially generated and real-world data by using the efficient implementation of set-trie written in the C programming language [28].

The set-trie is a tree data structure derived from the *trie* [29]. While tries are used to store and search words, i.e., sequences of symbols, the set-trie is used to store and query sets. The possibility to extend the performance of usual trie from the membership operation to the set containment operations comes from the fact that we are storing *sets* where the ordering of elements is not essential, while ordinary tries are used to store the *sequences* of symbols where the ordering of symbols is essential. The ordering of set elements and the representation based on the common prefixes provide the means for the definition of efficient algorithms for the set containment operations.

**Mathematical analysis**. In the mathematical analysis, the expected performance of the data structure is analyzed by a probabilistic model, similar to the one used in the related work by Rivest [30, 31] (i.e., we assume that the database content is generated uniformly at random). Standard tools such as Bernoulli distribution and Galton-Watson branching stochastic process are used. As a result, some relevant upper-bounds on the time-complexity are determined. Our approach provides efficient set containment operations where the time/space complexity is parametrized on the value of density |*S*|/2^|Σ|^, and also on other relevant parameters such as the cardinality of the input set *X* or the choice of elements in *X*.

In the dense case, i.e. when |*S*| = Θ(2^|Σ|^), the expected running time of existsSubset and existsSuperset turns out, asymptotically, to be of order *O*(1) and *O*(log|*S*|), respectively. For getAllSubsets and getAllSupersets we obtain asymptotically best possible running times, that is, linear in terms of the size of the output. In both theoretical as well as empirical analysis, we observe the following *element frequency ranking tradeoff*: for existsSubset and getAllSubsets the input sets with low-ranked members perform considerably faster than those with high-ranked ones. On the other hand, for existsSuperset and getAllSupersets the input sets with high-ranked members perform faster.

**Empirical analysis**. The empirical analysis comprises two parts. In the first part, we have used the artificially generated data to analyze the time complexity of the presented methods in three experiments. In the second part of the empirical analysis, we compare the performance of the set-trie with the inverted index. We used three different real-world datasets in experiments: the sets of sets generated by the data mining tool fdep, and two datasets storing the page requests of users from two different Web sites. One of these two datasets is skewed in the sense that the frequencies of the alphabet symbols in sets vary significantly.

In the first three experiments on the artificially generated data, we observe the influences of the selected parameters on the performance of the set containment operations. In Experiment 1, we investigate the influence of the size of set-trie on the performance of the set containment operations. We show the basic shapes and the experimental upper-bounds of the curves representing the number of visited nodes for all operations. The results of the subset and superset operations can be seen as dual. Experiment 2 gives some insight into the influence of the size of the alphabet on the behavior of operations. The curves for all operations scale almost linearly with the increasing size of the alphabet. In Experiment 3 we study the influence of the structure of the input sets on the performance of operations. The empirical analysis confirms the findings from the mathematical analysis. We show that the superset operations work much faster than the subset operations when the test-sets are composed of elements with low-frequency rank. In contrast, the subset operations outperform the superset operations when the test-sets contain elements with high-frequency rank. The subset and superset operations exhibit the dual behavior again, but in a stronger way than previously observed.

The three experiments on the real-world data compare the set-trie with the inverted index. The three datasets that we use in the experiments include the sets with low cardinality. Similarly to Helmer and Moercotte in [32], we also observe that most real-world application datasets include sets with low cardinality. The results of all three experiments show that the set-trie performs orders of magnitude better than the inverted index. In Experiment 4, we use the data mining tool fdep [26] to generate the sets of sets that represent the intermediate results in the computation of the functional dependencies from the input relations. Experiment 5 compares the performance of indexes on the msnbc dataset [33] storing the sequences of Web site areas accessed by the visitors of the msnbc Web site. The subset and superset existential containment operations performed extremely fast because of the high density of smaller sets in the domain. In Experiment 6, we compare the indexes on the msweb [33] dataset that stores page accesses for users of the microsoft.com Web site. The data is skewed since some areas of the Web site are visited much more frequently than others. The results of this experiment show that skewed data do not degrade the performance of set-trie operations. The main reason for this is in the explicit representation of sets in a set-trie based on the sorted sequences of the set elements and the common prefixes, which allows fast exploration of similar sets.

### 1.3 Contributions

This paper presents a thorough mathematical analysis and a detailed empirical analysis of the set-trie data structure that was introduced in [27]. The contributions of the presented research are as follows:

- The contributions of the mathematical analysis are the asymptotic estimates of upper-bounds of the time-complexity for the set containment operations.
- The presented experiments on the artificially generated data give a detailed insight into the structure of the search space of set containment operations as well as the experimental upper-bounds for all operations.
- The comparison of the set-trie with the inverted index on the real-world datasets storing sets with low cardinality shows that the set-trie outperforms the inverted index by orders of magnitude better running time.
- We obtained insight into the influence of ordering in the alphabet Σ on the performance of the set-trie. The ordering of symbols from an alphabet can be tuned for the specific application domain as well as for the selection of the set containment operations.
- Finally, the results of the experiments on skewed data show that the set-trie data structure is robust to the skewness.

### 1.4 Paper organization

The paper is organized as follows. In Section 2, we present the related work from the different areas of computer science. The related work from theoretical computer science, information retrieval, artificial intelligence, object-relational database systems, and Internet applications are described in Sections 2.1-2.5, respectively. Section 3 presents the data structure set-trie together with the set containment operations. We give a detailed presentation of the operations insert, existsSubset, existsSuperset, getAllSubsets and getAllSupersets and delete. Next, Section 4 presents the mathematical analysis of set-trie. The description of the mathematical model is given in Section 4.2. The estimation of the number of visited nodes in the subset operations is studied in Section 4.3. The estimation of the number of visited nodes of the superset operations is presented in Section 4.4. Section 5 describes an empirical analysis of set-trie. The first part of the empirical analysis based on the artificially generated data is presented in Section 5.1. The three experiments that study the influences of the selected features of set-trie on the time complexity of the set containment operations are presented in Sections 5.1.2-5.1.4. The second part of the empirical analysis is given in Section 5.2. We compare the set-trie to the inverted index on the real-world datasets in three experiments presented in Sections 5.2.2-5.2.3. Finally, the conclusions and the directions of our further work are given in Section 6.

## 2 Related work

The problem of querying sets of sets appears in the following areas of computer science.

**Algorithms and data structures**. The problem has been studied in the form of *partial-matching* in the area of algorithms by Rivest [34], Baeza-Yates [35], and Charikar [36]. There has been very little interest in this problem in the last two decades.
**Information retrieval**. In Information retrieval, the inverted index is a central data structure of an IR system. It stores documents as multisets of words and queries them by specifying words to be included in selected documents [2, 3]. Because of the important role of IR in the development of Internet search engines, the inverted files continue to attract significant research attention.
**Artificial intelligence**. The subset queries are studied in various sub-areas of artificial intelligence for storing and querying: pre-conditions of a large set of rules [10], states in planning for storing goal sets [8] and hypotheses in data mining algorithms [37]. There has been considerably less interest in data structures designed for fast set containment queries in data mining and in AI in general [9, 38] in the last two decades.
**Object-relational database systems**. Querying sets is an important problem in object-relational database management systems where attributes of relations can range over sets [5, 14, 39, 40]. The efficient implementation of the set containment join [13, 15–19] continues to be an active research problem.
**Internet applications**. Sets are used in Internet applications for the representation of the properties of objects, sparse vector data, text files, itemsets, tags and the neighbours in graphs. Two types of queries attracted research attention: the similarity search [20, 22] and the set similarity join [23–25, 41]

We present each of the above stated related areas in more detail in the following Sections 2.1-2.5.

### 2.1 Algorithms and data structures

The data structure we consider is similar to the data structure *trie* [29, 34]. A trie is a tree that stores sequences of symbols from a given alphabet as paths starting in a trie root and ending in a node marked as the end of the sequence. A trie makes use of the common prefixes of sequences to save space. Since we are not storing sequences but *sets* in the set-trie, we can exploit the fact that the order in sets is not important. Therefore, we can take advantage of this to use the syntactical ordering of the set elements in the design of the set containment operations.

Our problem is similar to the partial matching of strings for which the tries and Suffix trees can be used. Rivest examines [34] the problem of partial matching with the use of the hash functions and the trie trees. The algorithms based on hash functions and tries are analyzed. It has been shown that tree algorithms are approximately as efficient as the hash-based algorithms. They use time about *O*(*n*^(*k*−*s*)/*k*^) where *n* is the number of sets, *k* is the size of sets, and *s* is the number of fixed symbols of a pattern. Rivest does not use the ordering of indexes in trie as we do. This can only be done in the case that the sets or multisets are stored in tries where the ordering of set elements is not essential.

Baeza-Yates and Gonnet present an algorithm [35] for searching instances of regular expressions using Patricia trees as the logical model for the index. The prefixed regular expressions can express substrings as well as superstring operations. The algorithm converts a regular expression into a finite automaton that is employed for searching the expressions. The results of the search are the nodes of Patricia trees that represent subtrees of words. The main result of the work is the logarithmic expected time of the algorithm for a subset of regular expressions, and sublinear time for general regular expressions.

Finally, Charikar et al. [36] present two algorithms to deal with a subset query problem, similar to our operation existsSuperSet. The analysis of their algorithm is performed for the case when the collection of sets is sparse, i.e. its density |*S*|/2^|Σ|^ tends to 0. They show the following tradeoffs:

- $|S|\cdot 2^{O(|\Sigma |{\text{log}}^{2}|\Sigma |\sqrt{c/\text{log}|S|})}$ space, and *O*(|*S*|/2^*c*^) time, for any *c*;
- |*S*| ⋅ |Σ|^*c*^ space and *O*(|Σ||*S*|/*c*) query time, for *c* ≤ |*S*|.

In such sparse setting, the space/time complexity of our algorithm would be bounded above to min(|*S*| ⋅ |Σ|, 1.5^|Σ|^).

### 2.2 Information retrieval

The information retrieval (abbr. IR) area deals with storing and querying huge collections of text documents [1, 2, 4, 42]. The most common model used for the representation of texts is the bag-of-words model of documents. A document is represented by a multiset of words that are preprocessed using techniques such as stemming, stopping, lemmatization, and others. After the words of the documents are preprocessed, the inverted file is constructed to support a range of query types from simple word matchings to boolean queries typical for the early IR systems. The inverted file is the most important part of an IR system; it is an all-in-one data structure that serves as the structural framework for the representation of the data, and for querying the collections of texts.

The inverted file is composed of two main parts, the dictionary, and the postings. In the baseline inverted file, the dictionary is used to identify, for the given words, the postings that are composed of a list of document identifiers. In addition, postings usually include a pointer to the location of the word inside the document and the frequencies of words in documents. The data structures commonly used in inverted files for the representation of a dictionary are hash tables, B-trees, and tries [14]. The dictionary is typically stored in the main memory to speed up the access to postings. Because of the huge amount of texts stored by the IR system, the postings have to be stored on disks [2].

Inverted files support boolean queries. The conjunction of two or more one-word queries retrieves documents that include both words. The disjunction of one-word queries includes all documents listed for one-word queries. Finally, the negation of a one-word query includes all documents that do not include a given word. The boolean expressions can be arbitrarily combined by using operations conjunction, disjunction, and negation. The time complexity of query evaluation is *O*(*N*), where *N* is the number of documents in a collection. The efficiency of the query evaluation in practical IR systems depends on the careful implementation of the inverted file, and efficient methods for query evaluation. Let us present some optimizations of the baseline inverted file [2].

Since postings include the integer numbers solely, the space used for the representation of integer numbers can be significantly reduced by using variable-length encodings. The benefits of compression are a more efficient representation of data and faster access to postings. The disadvantage of compression is the need to decode the postings before they can be used in calculations. Next, the processing of the postings can be sped up by introducing additional structure in postings. A technique called *skipping* organizes postings into chunks that are used to guide the search during query evaluation. Further, it is beneficial that the postings are *sorted*, either by frequencies or by the actual impacts of words to the similarity measure. Only the most influential hits are interesting for further processing. Finally, the *ordering of postings* that are linked to the words from a query is very important for query processing. While the efficient order of processing the postings is an optimization problem, the greedy approach that selects the shortest lists first works well [1].

An alternative to the inverted file was proposed in the area of Information retrieval by Deppisch et al. in the form of a dynamically balanced signature tree [3]. Signatures are hash-coded binary words of a fixed length that represent abstractions of objects. Each object attribute value is mapped to a sequence of bits that are carefully defined to represent an abstraction of the attribute value. The bits representing attribute values are then glued together to form a signature. The main advantage of signatures used as the access paths in database systems is the possibility to express partial matching, subset queries, substring matching, and fuzzy match queries.

In the simplest instance, the *signature file* stores the signatures of objects solely. The search is implemented by a sequential scan of the signature file retrieving the signatures having the bits in the query set to one [43]. The search can be improved by structuring the signatures hierarchically. The signatures are not just the keys; they have additional information encoded into the signatures. We can decode from the signature the values of particular attributes of a stored object. Furthermore, given a set of signatures, they can be superimposed (computed using the bitwise OR operation) to obtain a representative signature of a given set of signatures. The signatures are therefore used to guide the search. Given a superimposed signature and a signature that represents an object, we can determine just from the signatures if the superimposed signature can represent a given concrete signature.

The dynamically balanced *signature tree*, called the S-tree, is in many aspects similar to B+-tree [3]. The S-tree is comprised of index pages and leaf pages. The index pages store lists of pairs composed of signatures and pointers to the corresponding subtrees. The signatures at the higher levels are constructed by superimposing the signatures from the corresponding subtrees. The leaf pages store pairs composed of signatures and, either objects or pointers to objects stored in some other file. An S-tree is a dynamically balanced tree; all leaf pages of S-tree have equal height.

The main operations of S-tree are *retrieve*, *insert*, *update* and *delete*. The operation *retrieve* follows the paths from the root to a leaf node that is determined by relating the signature to the signatures from index pages. The operation *insert* enters the new signature in a leaf page that is determined by the new signature. A leaf page is split into two pages if the number of signatures exceeds the maximal number of signatures. Splitting a page is implemented by grouping signatures by similarity into two pages. The operation *delete*, on the other hand, deletes the signature from the leaf page and updates the affected signatures in index pages. When the number of signatures on the page is lower than the minimum, then two nodes are joined into a single node. The operation *update* changes the value of a given signature and updates the affected signatures in index pages.

### 2.3 AI systems

The initial implementation of set-trie was in the context of a data mining tool fdep [26], which is used for the induction of functional dependencies from the relations [7, 44]. It has been further used in the data mining tool mdep [45] for the induction of multivalued dependencies from the relations. In both cases, the sets are used as the basis for the representation of dependencies. Hypotheses (dependencies) are checked against the negative cover of *invalid dependencies* represented by means of the data structure set-trie. Furthermore, the positive cover, including redundant valid dependencies, is minimized by using the data structure set-trie as well. The set-trie is related to the indexes proposed for storing sets of sets in various AI systems [8, 10, 11, 37] including the data mining tools for the discovery of functional dependencies from relations [9].

Doorenbos in [11] proposes an index structure for querying pre-conditions of rules to be matched while selecting the next rule to activate in a rule-based system Rete [10]. The index structure stores the conditions in separate nodes that are linked together to form the pre-conditions of rules. Common conditions of rules are shared among the rules: the lists of conditions with a common prefix share all nodes that form the prefix. Given a set of conditions that are fulfilled, all rules that contain as a pre-condition a subset of a given set of conditions can be activated.

An index data structure for storing the sets of sets is proposed by Hoffman and Koehler as Unlimited Branching Tree (abbr. UBTree) [8]. The main difference to the representation of rules in the expert systems is that UBTree does not use variables. The children of a node are stored in a list attached to the node. A set in UBTree is represented by a path from the root to the final node. The elements of the set label the path. The search procedures for the subset and superset problems are similar to those we propose; however, the main difference in procedures is that we explicitly use the ordering of sets for search while Hoffman and Koehler give a general algorithm allowing other heuristics to be exploited. Our publication in 1993 [44] evidently presents the independence of the work.

Mamoulis et al. propose in [37] the use of a balanced signature tree, called SG-tree, for storing sets in data mining applications. The architecture of the proposed SG-tree is very similar to a dynamically balanced signature tree S-tree [3]. An SG-tree is appropriate for storing sets of sets in a dynamic environment with frequent updates. They show experimentally that an SG-tree can be used for the similarity search.

### 2.4 Object-relational database systems

The sets are among the important data modeling constructs in the object-relational and object-oriented database systems. The *set-valued* attributes are used for the representation of properties that range over the sets of atomic values or objects. The database community has shown significant interest in indexing structures that can be used as the access paths for querying set-valued attributes [5, 13, 14, 39, 40]. *Set containment queries* were studied in the frame of different index structures.

Zhang et al. [40] investigated two alternatives for the implementation of the set containment queries: a) a separate IR engine based on the inverted lists, and, b) the native tables of the relational database management system. They have shown that while RDBMS is poorly suited for the set containment queries, they can outperform the inverted list engine in some conditions. Furthermore, they have shown that with some modifications, RDBMS can support containment queries much more efficiently.

Another approach to the efficient implementation of the set containment queries is the use of signature-based structures. Tousidou et al. [39] combine the advantages of two access paths: the linear hashing and the tree-structured methods. They show through the empirical analysis that an S-tree that uses linear hash partitioning is an efficient data structure for the subset and superset queries.

Helmer and Moercotte investigated four index structures for querying set-valued attributes of low cardinality [5]. All four index structures are based on conventional techniques: signatures and inverted files. The index structures that they compare are the sequential signature files, the signature trees, the extendable signature hashing, and B-tree based implementation of inverted lists. The inverted file index showed the best performance over the other data structures in most operations.

Terrovitis et al. [46] improve the performance of the inverted files by ordering the symbols (i.e., the set elements) from the vocabulary Σ. The sets are in an ordered inverted file (OIF) represented by using the ordered lists of elements. Therefore, the lexicographical ordering of sets can be defined. But then, the sets can be indexed in the same way as any other collection of key/value pairs by using B+ trees. The postings of an inverted file are stored in separate B+ trees that are merged in a single B+ tree index. The set containment operations are implemented efficiently by identifying the intervals of B+ index that are mapped to a range of disk blocks containing the candidate results. There are similarities between the OIF and the set-trie. The most evident similarity is in the ordering of the elements of sets. However, there are differences in the use of the ordering. A set-trie uses the ordering of sets for the space-efficient representation of the sets of sets. Further, the ordering of sets in a set-trie is employed for reducing the search space to a sub-tree of a set-trie. The search space is similarly reduced in the OIF. The difference is in the way a sub-tree of a given search tree (a set-trie, or an OIF B+ tree) is defined for a given query.

A number of join algorithms based on set containment operations are proposed [13, 15–17]. The partitioning set join [15] relies on the signature-based representation of the sets. The sets are abstracted by means of signatures to provide fast set comparison operations. The sets are converted to signatures by converting the set elements into the bits of the signatures using the modulo function. The partitioning set join [15] splits records of the input relations by using the values of the given set-valued attributes. In addition, partitioning eliminates unnecessary comparisons between signatures. The partitioning set join algorithm is further improved to handle efficiently large sets as well as to optimize the partitioning phase of the algorithm by the adaptive design of monotonic hash functions for particular relations [13].

Finally, Jampani and Pudi propose the use of a prefix tree together with an inverted index for the computation of the set-based joins of two relations [16]. The proposed set-based joins are referred to as PRETTI joins. The set containment join of the table *R* with the table *S* is computed by constructing a prefix tree for the sets from *R* and an inverted index for the sets from *S*. Starting with the root of the prefix tree constructed for *R*, the algorithm for the set containment join traverses the prefix tree in depth-first order. In each node *n* of the prefix tree, the list of rids of the records from *S* that contain a given set represented by *n* is computed. This can be done recursively by intersecting rids from *S*, computed previously for a common prefix, with the rids of records from *S* containing *n*. The algorithm is efficient since a single intersection of two lists is required to be computed to enumerate matching tuple pairs from *S* for a given set from *R*. Besides the algorithm for the set containment join, the paper proposes the algorithms for the set overlap join and the set equality join. The PRETTI set-based join algorithms proposed in [16] are further improved by Luo et al. [17] by using the Patricia trie instead of the prefix tree. The empirical results show that their PRETTI+ algorithm outperforms the state-of-the-art set-based joins by an order of magnitude.

### 2.5 Internet applications

The operations that are closely related to the set containment queries are the similarity search and the set similarity join. These two operations were recently studied in the Web environment [20] in the form of the query refinement for the Web search, and for finding near-duplicate documents [21]. The application areas that use some form of set similarity search operations are data cleaning, information integration, community mining and entity resolution [25]. Furthermore, the documents and queries in Internet applications are represented by multisets, i.e., sparse vectors in a high-dimensional space. The techniques from Information Retrieval, such as the Cosine and Jaccard similarity measures, can be employed for measuring the similarity of multisets [1].

Given two collections of sets, the *exact set similarity join* [23–25] computes all pairs for which the similarity measure function gives a value above the given threshold. The similarity measure in this approach is based on cumulative weights computed using indexing and optimization [20]. The proposed solution uses a filtering-verification framework where all pairs of sets are first filtered by various heuristic techniques and afterward verified to check if they meet the similarity threshold. The filtering techniques include prefix filtering [20], generalized prefix filtering [22], positional and suffix filtering [21], grouping and pruning [41], removing obsolete entries in inverted lists [47] and position-enhanced length filtering [48].

## 3 Data structure set-trie

Let a set of symbols {*s*_1_, *s*_2_, …, *s*_*σ*_} be an alphabet Σ of the size *σ*. We want to store a set of sets of elements from Σ efficiently. The data structure for storing the set of sets should provide efficient algorithms for the operation insert, the membership operation, and the set containment operations.

A syntactical order of symbols from Σ can be defined by assigning each symbol a unique index. The indexes are the elements of the set {1, 2, …, *σ*}. The assignment of the indexes to the symbols from Σ is used to obtain a unique representation of a set of symbols. It can be represented using a set of integer numbers. Furthermore, it turns out that a careful choice of the order of Σ can contribute to the efficient implementation of the set containment operations.

To simplify the presentation of the data structure set-trie, but without any loss of generality, we assume that Σ = {1, …, *σ*}. Since the elements of Σ are totally ordered, we can exploit this ordering for the efficient representation of a set of sets, as well as for the design of efficient algorithms for the set containment operations. Therefore, a set can be represented by an ordered sequence of integer numbers from Σ. This is the representation of sets that we use for storing sets in the data structure set-trie.

The data structure *set-trie* is a tree composed of nodes labeled with indexes from 1 to *σ*. It is structured as follows:

- The root node is labeled with ∅, and its children can be the nodes labeled from 1 to *σ*. A root node alone represents an empty set.
- A node labeled *i* can have children labeled with the indexes that are greater than *i*.
- Each node can have a flag *last_flag* denoting the last element in the set.

**Fig 1:**
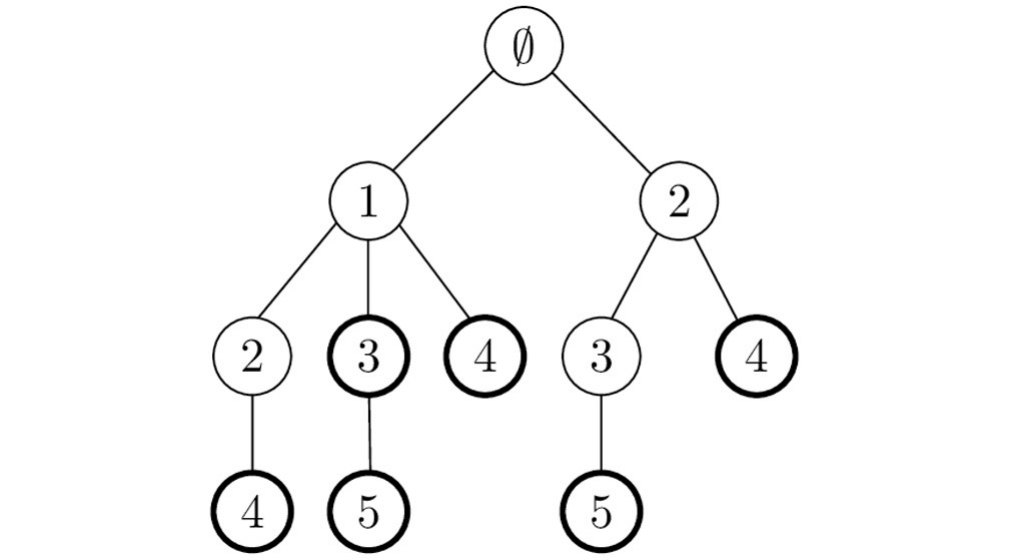
An example of a set-trie.

A set is represented by a path from the root node to a node with *last_flag* set to true. Note that the path is composed of nodes labeled by the indexes that are increasing along the path. Let us give an example of a set-trie. Fig 1 presents a set-trie containing the sets {1, 3}, {1, 3, 5}, {1, 4}, {1, 2, 4}, {2, 4} and {2, 3, 5}. Note that flagged nodes are represented with circles.

Let *S* be a set-trie, *X* a set of indexes from Σ, and, *L* an ordered list of indexes from *X* such that *L*[1] stores the smallest index and *L*[|*X*|] holds the highest index from *X*. A set *X* is in a set-trie *S* represented by a path *p* from the root of *S* to some node that has the flag *last_node* set to true. The path *p* is composed of the nodes labeled by the indexes that correspond to the elements of the ordered list *L*. The prefixes that overlap are represented by a common path from the root to an internal vertex of set-trie tree.

The operations for searching the subsets and supersets of a set *X* in a set-trie *S* employ the ordering of indexes. The algorithms do not need to consider the tree branches which we know do not lead to the results. The search space for a given *X* and a set-trie *S* can be seen as a sub-tree, or, a strip of the set-trie *S* of the size that depends on the set *X* and the set-trie *S*.

### 3.1 Operations on sets

Let *X* ⊆ Σ and *S* be a set-trie that represents a collection of sets *W* = {*s*_*i*_|*s*_*i*_ ⊆Σ}. To simplify the presentation, we write *X* ∈ *S* if *X* ∈ *W*. We are interested in the following operations:

1. insert(*S*, *X*) inserts the set *X* into the set-trie *S*;
2. search(*S*, *X*) returns *true* if *X* ∈ *S* and *false* otherwise;
3. existsSubset(*S*, *X*) returns *true* if ∃*Y* ∈ *S*: *Y* ⊆ *X* and *false* otherwise;
4. existsSuperset(*S*, *X*) returns *true* if ∃*Y* ∈ *S*: *X* ⊆ *Y* and *false* otherwise;
5. getAllSubsets(*S*, *X*) returns all sets *Y* such that *Y* ∈ *S*∧*Y* ⊆ *X*;
6. getAllSupersets(*S*, *X*) returns all sets *Y* such that *Y* ∈ *S*∧*X* ⊆ *Y*; and
7. delete(*S*, *X*) deletes the set *X* from the set-trie *S*.

Let us now present the data structure for storing sets that will be used for the presentation of the operations. Since the sets of indexes from Σ that we use in the algorithms rely on the ordering of indexes, we need a special data structure for the representation of sets. The data structure *Set* is represented as a class in an object-oriented programming language: it has a state and a set of operations.

The state of the *Set* instances includes the *current element* of the set. The current element is a concept similar to the file pointer that points to some position in a file. Analogously, the current element of a set is an element of the set that we currently observe. Let *X* ⊆ Σ and *c* ∈ *X* is the current element of a set *X*. The *next element* of *c* is the smallest *n* such that *n* ∈ *X* and *c* < *n* with respect to the ordering of Σ.

Let *X* denote an instance of the class *Set*. The class *Set* has the following methods. The method *X*.first() sets the current element of the set to the element of *X* that has the smallest value. The operation *X*.existsCurrent() checks if there exists the current element in the set *X*. The method *X*.current() returns the current element of the set *X* if it exists and returns an error otherwise. The method *X*.next() returns the next element of the current element, and, *σ* + 1 if there is no such element. The current element is not defined after the method next() is applied to the set *X*, where the current element is the last (the greatest) element of *X*.

**Algorithm 1** Procedure insert(*V*, *X*)

1: **procedure** insert(*V*, *X*)

2:  **if** *X*.existsCurrent() **then**

3:   **if exists** child of *V* labeled *X*.current() **then**

4:    *U* ← **retrieve** child of *V* labeled *X*.current();

5:   **else**

6:    *U* ← **create** child of *V* labeled *X*.current();

7:   insert(*U*, *X*.next())

8:  **else**

9:   *V*.last_flag = *true*

### 3.2 Operation insert

The first operation of set-trie data structure is *insertion*. The operation insert(*V*, *X*) enters an instance *X* of the type *Set* into the set-trie *S* referenced by a root node *V*. The operation insert is presented in Algorithm 1. Each invocation of the operation insert either traverses through the existing tree nodes or, creates the new nodes to construct a path from the root to the flagged node corresponding to the last element of the set *X*.

**Algorithm 2** Function search(*V*, *X*)

1: **function** search(*V*, *X*)

2:  **if** *X*.existsCurrent() **then**

3:   **if exists** child of *V* labeled *X*.current() **then**

4:    *U* ← child of *V* labeled *X*.current()

5:    search (*U*, *X*.next())

6:   **else return** *false*

7:  **else return** *V*.last_flag

### 3.3 Operation search

The operation search(*V*, *X*) searches for a given *X* in a set-trie *S* represented by a tree with the root *V*. It returns *true* if *X* is an element of the set-trie represented by *V*, and, *false* otherwise. The operation search is presented in Algorithm 2. As in the case of ordinary *trie* data structure, operation search checks if there exists a path from the root of tree *V*, labeled with the elements (indexes) of *X*, to some node flagged as *last_node*.

Let us give some more details about the algorithm of the operation search. The operation has to be invoked with the call search(*V*, *X*.first()) so that *V* is the root of the set-trie tree and the current element of *X* is the smallest index of *X*. Each activation of search tries to match the current element of *X* with the child of *V*. If the match is not successful, it returns *false*; otherwise, it proceeds with the following elements of *X*.

### 3.4 Operations existsSubset and getAllSubsets

The operation existsSubset(*V*, *X*) checks if there exists a subset of *X* in the given set-trie *S* with the root *V*. The subset that we search in *S* can have fewer elements than *X*. Besides searching for the exact match, we can also skip one or more elements in *X* and find a subset that matches the rest of the elements of *X*. The operation is presented by Algorithm 3.

**Algorithm 3** Function existsSubset(*V*, *X*)

1: **function** existsSubset(*V*, *X*)

2:  **if** *V*.last_flag **then**

3:   **return** *true*

4:  **if not** *X*.existsCurrent() **then**

5:   **return** *false*

6:  *found* ← *false*

7:  **if exists** child of *V* labeled *X*.current() **then**

8:   *U* ← child of *V* labeled *X*.current()

9:   *found* ← existsSubset(*U*, *X*.next())

10:  **if** *found* **then**

11:   **return** *true*

12:  **else**

13:   **return** existsSubset(*V*, *X*.next())

In the initial state of the algorithm, the parameter *X* has a current value set to the first element of the set, and the parameter *V* references the root of set-trie. The operation existsSubset tries to match elements of *X* with the child nodes of the set-trie *V*. In each step, either the current element of the *X* can be matched with a child of *V*, or the current element of *X* is skipped, and the operation tries to match the next element of *X* with the same set-trie *V*.

The first if statement in line 2 checks if a subset of *X* is found in the tree, i.e., the current node of a tree is the last element of the subset and last_flag = *true*. The second if statement in line 4 checks if *X* has no more elements, and, we did not find the subset in *V*. The third if statement in line 7 verifies if the parallel descends in *X* and in the tree *V* is possible. In the positive case, the algorithm calls existsSubset with the next element of *X* and the child of *V* corresponding to the matched symbol. Finally, if the match did not succeed, the current element of *X* is skipped, and existsSubset is called with the same *V* and the next element of *X* in line 13.

The operation existsSubset can be easily extended to find all subsets of a given set *X* in a tree with the root *V*. After finding the first subset in line 3, it must be stored, and the search can continue. In addition, instead of checking if a subset has already been found in lines 10-13, the operation getAllSubsets(*V*, *X*.next()) would be called to collect all the results. The experimental results with the operation getAllSubsets(*V*, *X*) are presented in Section 5.

**Algorithm 4** Function existsSuperset(*V*, *X*)

1: **function** existsSuperset(*V*, *X*)

2:  **if not** *X*.existsCurrent() **then**

3:   **return** *true*

4:  *found* ← *false*

5:  *element* ← *X*.current()+1

6:  *nextElement* ← *X*.next()

7:  **while** *element* ≤ *nextElement* **and not** *found* **do**

8:   **if exists** child of *V* labeled *element* **then**

9:    *U* ← child of *V* labeled *element*

10    **if** *element* = *nextElement* **then**

11:     *found* ← existsSuperset(*U*, *X*.next())

12:    **else**

13:     *found* ← existsSuperset(*U*, *X*)

14:   *element* ← *element* + 1

15:  **return** *found*

### 3.5 Operations existsSuperset and getAllSupersets

The operation existsSuperset(*V*, *X*) checks if there exists a superset of *X* in the set-trie *S* referenced by the node *V*. In operation existsSubset, we could skip some elements from *X* to match the sets from the set-trie *S*. In operation existsSuperset, we can do the opposite: we can omit some elements in the supersets from *S* to match the parameter set *X*. This operation is presented in Algorithm 4.

Let us present the algorithm of the operation existsSuperset in more detail. The initial state of the operation is the current element of the parameter set *X* is set to the first element, and the parameter *V* stores the reference to the root of set-trie *S*. In each recursive step, the algorithm can either descend to the next current element of *X* and to the matched child node of set-trie *V*, or it can leave the set *X* unchanged and descend only in the set-trie referenced by *V* to the selected child node.

The first if statement in line 2 checks if we are already at the end of *X*. If this is the case, then the parameter *X* is covered completely with a superset from the set-trie referenced by root *V*. The code in lines 6-7 sets the interval of elements (lower and upper bounds) that can be used in the search for the supersets from *S*. Note that the first element of the interval is the current element of *X* plus 1, and the last element of the interval is the next element of *X*. In each pass of the while loop in line 7, we either descend in parallel in line 11 on both *X* and the set-trie referenced by *V*, in the case that we reach the upper bound of the interval, or, we take the current child and call existsSuperset on unchanged *X* in line 13. Of course, we can descend to the child node of *V* only in the case that the child exists, which is checked in line 8.

As in the case of the operation existsSubset, the operation existsSuperset can be extended to retrieve all supersets of a given set *X* in a tree with the root *V*. After *X* is matched completely in line 3, there remains a subtree of trailers corresponding to a set of supersets that subsume *X*. This subtree is rooted in a tree node, let say *U*_*k*_, which corresponds to the last element of *X*. Therefore, after *U*_*k*_ is matched against the last element of the set *X* in line 3, the complete subtree has to be traversed to retrieve all supersets that go through the node *U*_*k*_.

### 3.6 Operation delete

The last operation of the set-trie is the operation delete(*V*, *X*) presented in Algorithm 5. The operation deletes the set *X* from the set-trie *S* referenced by a root node *V*. To simplify the algorithm, we suppose that the set *X* is stored in the set-trie *S*. The operation delete traverses from the root to some node of *S* by following the elements of the set *X*. When the operation delete is returning from the recursion, it deletes the nodes that do not serve to represent the remaining sets from *S*. More precisely, the operation deletes those nodes from the path in *S* defined by *X* that do not have children and are not labeled as the final nodes with last_flag set to *true*.

**Algorithm 5** Procedure delete(*V*, *X*)

1: **procedure** delete(*V*, *X*)

2:  **if** *X*.existsCurrent() **then**

3:   *U* ← child of *V* labeled *X*.current()

4:   delete(*U*, *X*.next())

5:   **if not exists** child of *U* **and not** *U*.last_flag **then**

6:    **dispose** *U*

7:  **else**

8:   *V*.last_flag ← *false*

## 4 Mathematical analysis of running-time

In this section, we provide a mathematical analysis of the running time of the presented algorithms. In Section 4.1, we start by describing some basic structural properties of the proposed data structure. Before moving on with the analysis, in Section 4.2, we describe some additional notations and introduce certain initial assumptions of our model regarding the type and distribution of the input. In particular, our analysis is based on a strong assumption that all words are present in our collection of sets with uniform probability.

The core building block, which plays an important role in the expected performance of all four functions, is the initial cardinality of our set-trie, which we describe in Section 4.2.1. In the process, we use the idea of the Galton-Watson branching stochastic process, where we grow our population by consecutively attaching letters of our alphabet to our set-trie. We upper-bound the growth function for any member of the alphabet in Lemma 2, and later construct a probability generating a function that majorizes the probability distribution of the cardinality of our set-trie. Finally, we measure the expectation of this probability generating function by the standard derivation approach and obtain the desired expected cardinality in Proposition 7. For more resources on the Galton-Watson stochastic process, and on the applications of probability generating functions we refer the reader to [49], and to Chapters XI and XII of [50], respectively.

In Section 4.3, we observe the exponential behavior of the getAllSubsets function, while determining an additional upper-bound for the existsSubset function. The result of Section 4.2.1 is not only an answer for getAllSubsets (with different parameters, see Section 4.3.1), but is also an important upper-bound for the later analysis of existsSubset. In particular, we determine that in our model, the distribution of the running time of existsSubset is a majorization of a geometric distribution with parameter *p*.

In the Section 4.4, we observe that for method getAllSupersets, input sets with low-ranked members perform faster than those with high-ranked ones. The nature of the algorithm shows that the length of input may be as important as the label of its last character—hence, the problem is translated to the bound from Section 4.2.1 (again, with different parameters, see Section 4.4). For the function existsSuperset, we observe that as long as *p* and *α*_*k*_ are not very small and fairly large, respectively, the expected number of visited nodes will not be large. The bound is attained by carefully constructing appropriate Bernoulli variables, which allow us to construct geometric distribution. Such geometric distribution will again minorize the actual running-time distribution of existsSuperset. Since this last analysis may be a bit abstract to get a proper performance feeling, we conclude the section by a short discussion on the results.

### 4.1 Some properties of set-trie

Given the size of the alphabet *σ*, let us define *a complete set-trie* to be a set-trie corresponding to the whole power-set P([0,σ-1]) and denote the corresponding tree with $T_{\sigma }$. Clearly, any set-trie with the alphabet-size *σ* will correspond to some subtree of $T_{\sigma }$. Note that the tree $T_{\sigma }$ is a binomial tree. We now give some basic properties of the complete set-tries.

**Observation 1** *Let* $T_{\sigma }$ *be a complete set-trie on alphabet of size σ. Then the following holds*:

1. *The tree* $T_{\sigma }$ *contains* 2^*σ*^ *vertices*.
2. *All σ neighbors of the root induce subtrees that are isomorphic to* $T_{0},T_{1},T_{2},\ldots ,T_{\sigma -1}$.
3. *There is precisely* 2^*i*−1^ *nodes with label i, for* 1 ≤ *i* ≤ *N*.

**Fig 2:**
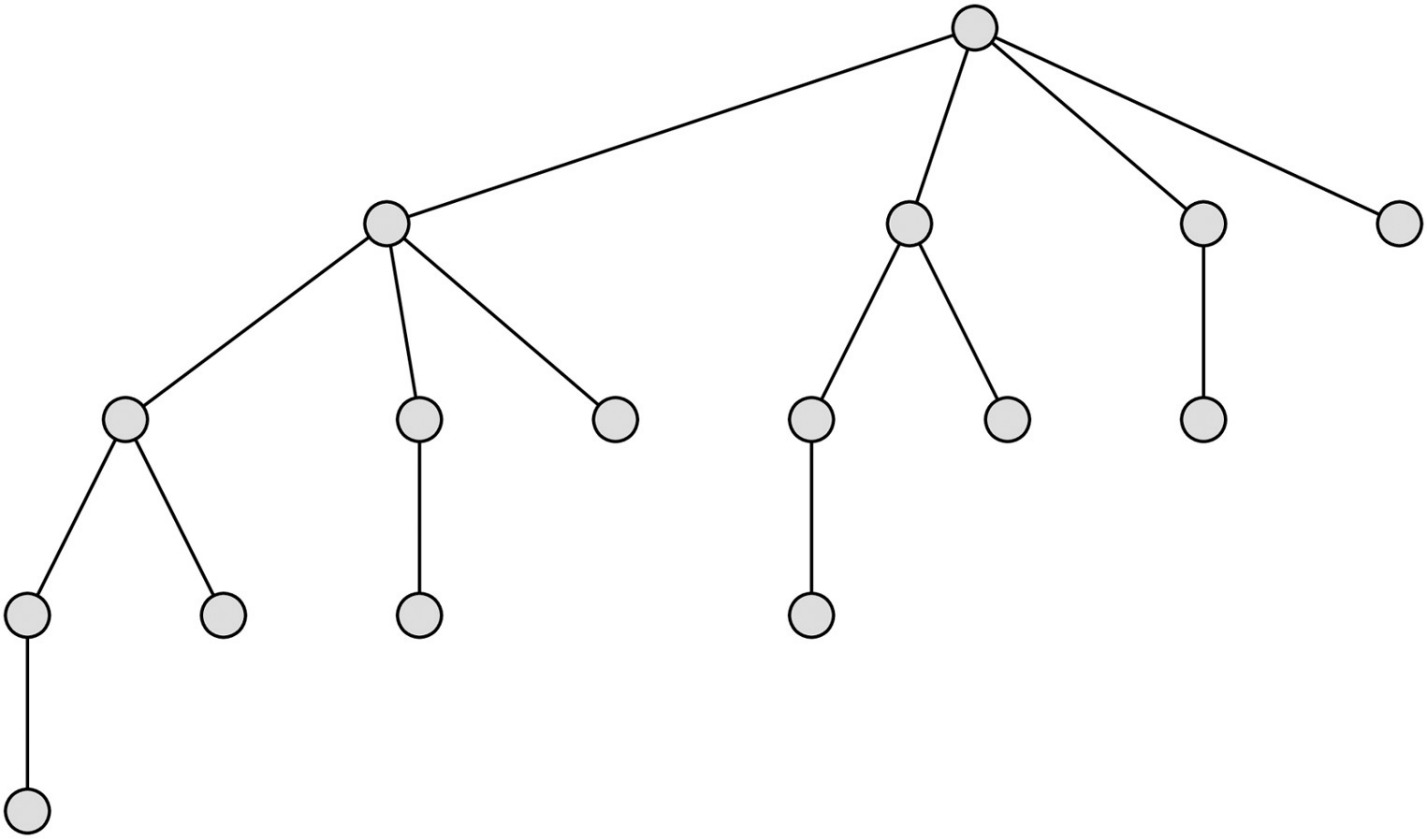
A complete trie for *σ* = 4.

For better illustration of a complete set-trie structure, see Fig 2 depicting the rooted tree that corresponds to a maximal set-trie, with *σ* = 4.

### 4.2 The description of the model

Let Σ be the alphabet of size *σ* and let *W* be a collection of sets from 2^Σ^ (i.e. subsets of Σ) stored in the corresponding set-trie *S*. By the implementation of the set-trie data structure, note that *S* can be interpreted as a subtree of $T_{\sigma }$, where each node contains a non-unique label *l* ∈ Σ. In this sense, observe that |*V*(*S*)| ≥ |*W*|, as for a given *w* ∈ *W*, the set-trie *S* also includes all prefixes of *w*.

For a fixed probability *p* ∈ (0, 1) and for *q* = 1 − *p*, our model assumes that for each set *w* ⊆ Σ we have *P*[*w* ∈ *W*] = *p*. Note that the expected number of sets stored in *W* equals to *p* ⋅ 2^*σ*^. For simplicity, we assume that Σ = {1, 2, …, *σ*}. For any subset *Y* ⊆ Σ we denote *S*[*Y*] the corresponding subgraph of *S*, induced by all *i*-labeled vertices, where *i* ∈ *Y*.

By Observation 1, there are 2^*i*−1^ vertices with label *i* in a complete set-trie $T_{\sigma }$, but most likely not all will be present in *S*. For any particular *i*-labeled node *v* from $T_{\sigma }$, let *p*_*i*,*σ*_ be the probability that *v* ∈ *S*, where *S* is generated by our model, on parameters *σ* and *p*. Observe that *v* is a root of a copy of $T_{\sigma -i}$, hence *v* ∈ *S* if and only if any word corresponding to members of its subtree is also a member of *W*. It is hence clear that

$$\begin{array}{ccc} p_{i,\sigma } & = & 1-\prod_{v\in T_{\sigma -i}}(1-p)=1-q^{2^{\sigma -i}}. \end{array}$$

Let *X* = *α*_1_ *α*_2_⋯*α*_*k*_ be a user specified input set consisting of *k* members of Σ. Our goal is to estimate an average time complexity for the method existsSuperset(*S*, *X*), getAllSupersets(*S*, *X*), existsSubset(*S*, *X*) and getAllSubsets(*S*, *X*). In order to get a proper insight of the model used, observe the following estimation of the expected number of nodes in *S*.

#### 4.2.1 The size of |*S*|

As we observed, it is easy to estimate the cardinality of *W*. In what follows, we try to upper-bound the value of |*S*|. The result, as well as the calculation, will help us later in complexity analysis of the above-mentioned methods. We start by calculating the conditional probability that, if *x*_*i*_ ∈ *S*, then *x*_*i*_ contains a neighbor labeled *i* + 1, which is denoted by *x*_*i*+1_.

**Lemma 2** *Let i, j, i* < *j be positive integers and let x_i_ be an arbitrary but fixed i-labeled vertex from S. The probability that it contains a j-labeled neighbor x_j_ under the assumption that x*_*i*_ ∈ *S can be upper-bounded to*

$$\begin{array}{c} P[x_{j}x_{i}\in E\left(S\right)|x_{i}\in S]\leq (1+q^{2^{\sigma -j}}{)}^{-1}, \end{array}$$

*with equality if and only if j* = *i* + 1.

**Proof 3** *By definition of conditional probability, we have*

$$\begin{array}{ccc} P[x_{j}\in S|x_{i}\in S] & = & \frac{P[x_{j}\in S\;\wedge \;x_{i}\in S]}{P[x_{i}\in S]}\\ & = & \frac{P[x_{j}\in S]}{1-q^{2^{\sigma -i}}}\\ & = & \frac{1-q^{2^{\sigma -i}}+q^{2^{\sigma -i}}-q^{2^{\sigma -j}}}{1-q^{2^{\sigma -i}}}\\ & = & 1-q^{2^{\sigma -j}}\frac{1-q^{2^{\sigma -j}\left(2^{j-i}-1\right)}}{1-q^{2^{\sigma -i}}}\\ & \leq & 1-q^{2^{\sigma -j}}P[x_{j}\in S|x_{i}\in S]. \end{array}$$

*By isolating the term P*[*x*_*j*_ ∈ *S*|*x*_*i*_ ∈ *S*] *from the expression above, the claim follows*.

For each character *i* ∈ Σ we now measure the size of a subtree of *S*, induced on all instances of vertices, with labels smaller than or equal to *i*. In particular, let *f*_*i*_ be a probability generating function for the corresponding distribution of the subtree size, i.e.

$$\begin{array}{c} f_{i}\left(x\right)=\sum_{j=0}^{2^{i}}P[|S[1,\cdots ,i]|=j]\cdot x^{j}. \end{array}$$

Let us state the standard definition of a majorizing sequence.

**Definition 4** *Let A* = *a*_1_, *a*_2_, …, *a*_*d*_ *and B* = *b*_1_, *b*_2_, …, *b*_*d*_ *be two number sequences, such that* $\sum_{i=1}^{d}a_{i}=\sum_{i=1}^{d}b_{i}$. *We say that A* majorizes *B, if* $\sum_{i=k}^{d}a_{i}\geq \sum_{i=k}^{d}b_{i}$, *for each k* ∈ [1, *d*].

We now define a generating function

$$\begin{array}{c} g_{i}\left(x\right)=\sum_{j=0}^{2^{i}}a_{i,j}x^{j}, \end{array}$$

with *g*_0_(*x*) = *x* which is recursively defined as *g*_*i*+1_(*x*) = *g*_*i*_(*p*_*i*_(*x*)), where $p_{i}\left(x\right)=\frac{x^{2}+x\;q^{2^{\sigma -i}}}{1+q^{2^{\sigma -i}}}.$ Function *g*_*i*_(*x*) is conveniently defined, as its coefficients majorize these from *f*_*i*_(*x*), as observed in the following claim.

**Lemma 5** *For each i* ∈ [1, *σ*] *and k* ∈ [0, 2^*i*^], *we have*

$$\begin{array}{c} \sum_{j=k}^{2^{i}}a_{i,j}\geq P[|S[1,\cdots ,i]|\geq k], \end{array}$$

*i.e. the coefficients from g_i_ majorize these from f*_*i*_.

**Proof 6** *We prove the claim by induction on i. For i* = 1, *we have* $g_{1}\left(x\right)=\frac{x^{2}+x\cdot q^{2^{\sigma }}}{1+q^{2^{\sigma }}}$, *while f*_1_(*x*) = *px*^2^ + *qx. Now suppose that the coefficients from g_i_ majorize these from f_i_, and observe that a probability generating function g*_*i*+1_ = *g*_*i*_(*p*_*i*_(*x*)) *represents some distribution obtained by a modified Galton-Watson process, with dynamic growth function* $p_{i}\left(x\right)=\frac{x^{2}+x\;q^{2^{\sigma -i}}}{1+q^{2^{\sigma -i}}}$. *From Claim 2 it is clear that the above-mentioned growth function actually upperbounds the actual growth of coefficients of f*_*i*+1_, *hence the conclusion*.

**Proposition 7** *The expected size of S is at most*

$$\begin{array}{c} \prod_{i=0}^{\sigma -1}[\frac{2+q^{2^{\sigma -i-1}}}{1+q^{2^{\sigma -i-1}}}]. \end{array}$$

**Proof 8** *From Lemma 5 it is clear that for any i, the distribution given by probability generating function f*_*i*_ *majorizes the one given by g*_*i*_. *Note that for any probability generating function* F(x), *it is well known that the expectation of the corresponding distribution equals to* $\frac{\partial }{\partial x}F^{\prime }(x){|}_{x=1}$ *(For detailed survey on probability generating functions we refer the reader to the book by Johnson et. al*. [51].*). As pointed out in Lemma 5, note that functions* {*g*_*i*_}_*i*≥0_ *are generated in a way similar to the Galton-Watson process, apart from the fact that in our case the reproduction function is not constant throughout the process, but changes at every step, and is given by* $p_{i}=\frac{x^{2}+x\;q^{2^{\sigma -i}}}{1+q^{2^{\sigma -i}}}$ for *i*-th generation. Furthermore, *g*_*i*_ *may be expressed as*

$$\begin{array}{c} g_{i}(x)=g_{i-1}(p_{i-1}\left(x\right))=p_{0}\circ p_{1}\circ \cdots \circ p_{i-1}(x), \end{array}$$

*while by the composition rule, its derivation is*

$$\begin{array}{c} g_{i}^{\prime }(x)=\prod_{j=0}^{i-1}p_{j}^{\prime }\circ \cdots \circ p_{i-1}(x). \end{array}$$

*The proof of the claim is concluded by*

$$\begin{array}{ccc} E(|S|)=f_{\sigma }^{\prime }(x){|}_{x=1} & < & g_{\sigma }^{\prime }{\left(x\right)|}_{x=1}\\ & = & \prod_{j=0}^{i-1}p_{j}^{\prime }\circ \cdots \circ p_{i-1}(x){|}_{x=1}\\ & = & \prod_{i=0}^{\sigma -1}p_{i}^{\prime }{\left(x\right)|}_{x=1}\\ & = & \prod_{i=0}^{\sigma -1}[\frac{2+q^{2^{\sigma -i-1}}}{1+q^{2^{\sigma -i-1}}}], \end{array}$$

*where the third line follows by the fact that p*_*i*_(1) = 1 *and* $p_{i}^{\prime }{\left(x\right)|}_{x=1}=\frac{2+q^{2^{\sigma -i-1}}}{1+q^{2^{\sigma -i-1}}}$, *for any admissible i*.

### 4.3 Analysis of subsets

In this section, we analyze the method regarding the subset queries. For getAllSubsets(*S*, *X*), it is clear that the length of the output is exponential in *k*. Hence we expect the obtained upper-bound also to be exponential.

In the case of exitsSubsets(*S*, *X*), the situation turns out to be more complex. Our upper-bound consists of two functions; one being useful for big densities of our set-trie, and the second one for sparse cases.

#### 4.3.1 Analysis of getAllSubsets(*S*, *X*)

When traversing the set-trie *S*, the algorithm visits only nodes in which labels appear in *X*. These nodes represent a subtree of depth at most *k* in *S*. We denote this tree with *S*[*X*], and let *w*_*X*_ be its size. In light of Proposition 7, it is easy to give an upper estimate of $E(w_{X})$ in the same way as we did with *S*, hence

$$\begin{array}{ccc} E(w_{X}) & \leq & \prod_{i\in X}[\frac{2+q^{2^{\sigma -i-1}}}{1+q^{2^{\sigma -i-1}}}]. \end{array}$$

As expected, since we do not assume that the input word *X* is chosen uniformly at random, the result shows that the expected running time in our model is highly input-sensitive. Observe that knowing the relative frequency of characters in a given input would allow us to choose the initial total ordering of the alphabet Σ in a correct way. In particular, the least frequent characters should be ranked higher than others. Indeed, observe that in real-world applications, *X* may be efficiently modeled by various randomized generations, such that given a character *c* ∈ *X* one may assume any of the following:

- *c* is chosen uniformly at random from [1, *σ*],
- *c* ∈ [1, log *σ*], with high probability,
- *c* ∈ [*σ* − log *σ*, *σ*], with high probability.

The trivial upper bound of *w*_*X*_ is 2^k^, which is attained at *p* = 1. Also, the reader should observe that the obtained bound is exponential in *k*, i.e. for any input word *X* there exists a constant *α* ∈ [1.5, 2], such that $E(w_{X})=\alpha^{k}$. Finally, the reader should realize that for most of the randomized models of *X* (including those mentioned above), it is easy to see that *α* → 2 when *σ* → ∞.

#### 4.3.2 Analysis of existsSubset(*S*, *X*)

In contrast to getAllSubsets(*S*, *X*), when traversing the tree *S*[*X*], the method existsSubset(*S*, *X*) stops at the first found flagged node. At any of the vertices in *S*[*X*], the algorithm stops with probability *p* or continues otherwise. First note that in the case when the result of existsSubsets(*S*, *X*) is False, then the complexity of existsSubset(*S*, *X*) in fact equals the complexity of getAllSubsets(*S*, *X*), analyzed in Section 4.3.1, and hence takes precisely *w*_*X*_ time steps. However, if this is not the case, we point out that the number of steps is distributed according to a geometric probability distribution on parameter *p*.

To be more precise, the probability of stopping at *i*-th visited node is *p* ⋅ (1 − *p*)^*i*−1^, for *i* ≤ *w*_*X*_. After searching through all possible sub-sets (i.e. after *w*_*X*_ steps), the algorithm stops—the probability of this scenario is clearly $(1-p{)}^{w_{X}}$. In particular, let X be a random variable that measures the number of steps made when running existsSubset(*S*, *X*). The value of P[X=t] is defined as follows:

$$\begin{array}{c} P[X=t]=\{\begin{array}{cc} p\cdot (1-p{)}^{t-1} & \;\text{if}\;1\leq t<w_{X};\\ p\cdot (1-p{)}^{w_{X}-1}+(1-p{)}^{w_{X}} & \;\text{if}\;t=w_{X}. \end{array} \end{array}$$

It is easy to check that this is a proper probability space that sums to 1, and that the probability space is very similar to a geometric distribution with parameter *p* (where the end of the right tail is cut off at *w*_*X*_). While *w*_*X*_ is still the upper-bound of T (existsSubset(*S*, *X*)), calculating an expectation of X gives us another upper-bound.

**Theorem 9** The expected number of steps of existsSubset(*S*, *X*) is equal to:

$$\begin{array}{c} \frac{1}{p}\cdot (1-(1-p{)}^{w_{X}}). \end{array}$$

In particular, the expected number of steps is at most $\frac{1}{p}$.

**Proof 10** *We calculate the expectation of the random variable* X,

$$\begin{array}{ccc} E(X) & = & \sum_{i}i\cdot P[X=i]\\ & = & w_{X}(1-p{)}^{w_{X}}+p\cdot \sum_{i=0}^{w_{X}}i\cdot (1-p{)}^{i-1}. \end{array}$$ (1)

*Setting q* ≔ 1 − *p*, *we get a closed form by integrating*

$$\begin{array}{c} \int (\sum_{i=0}^{w_{X}}i\cdot q^{i-1})dq=\frac{1-q^{w_{X}+1}}{1-q}+C. \end{array}$$

*Plugging this back into* (1), *we get*

$$\begin{array}{ccc} E(X) & = & w_{X}q^{w_{X}}+\left(1-q\right)\cdot \frac{d}{dq}(\frac{1-q^{w_{X}+1}}{1-q}+C)\\ & = & w_{X}q^{w_{X}}+\frac{w_{X}q^{w_{X}+1}-\left(w_{X}+1\right)q^{w_{X}}+1}{1-q}\\ & = & w_{X}q^{w_{X}}+\frac{1-q^{w_{X}}(w_{X}-w_{X}q+1)}{p}\\ & = & w_{X}q^{w_{X}}(1-\frac{1-q}{p})\\ & = & \frac{1}{p}(1-(1-p{)}^{w_{X}}). \end{array}$$ (2)

It is easy to see that the bound above is quite efficient in some cases—here we point out the two efficient situations. In the Example 11 we have a large alphabet comparing to the length of an input.

**Example 11** *Assume that our input is very short, i.e. k* = *o*(log *σ*). *Then we have*

$$\begin{array}{c} E(X)\leq w_{X}\leq 2^{k}\leq o(\sigma ). \end{array}$$

In the Example (ii) we have a dense set-trie comparing to the length of an alphabet.

**Example 12** *Suppose that our set-trie is quite dense, i.e. if* $p>\epsilon \cdot (\frac{1}{\sigma }),$ *for arbitrary small constant ϵ* > 0. *Then clearly* $E(X)\leq \frac{1}{p}<O\left(\sigma \right)$. *We believe that many of the real-world datasets are* dense *with respect to the criteria above. For example, an actual set of all words in English dictionary consists of approximately a million words, where a standard 26-letter English alphabet is used. Setting the appropriate values with σ* = 26, *p* = 0.0148 *and ϵ* = 0.385, *we expect the number of sets visited by* existsSubset(*S*, *X*) *to be bounded around 68 or less, or even less when*|*X*| *is small*.

While the analysis above mostly focuses on the expectation of the running time, observe that the distribution of X is well-concentrated around its expectation—for instance, even Markov inequality implies w.h.p. that X does not exceed its expectation by a factor of log *σ*, i.e.

$$\begin{array}{c} P[X>E(X)\;\text{log}\;\sigma ]\leq \frac{1}{\text{log}\;\sigma }\underset{w.h.p.}{⟶}0. \end{array}$$

Since in our model all sets are chosen with uniform probability *p*—resembling a geometric distribution, the type of the traversal algorithm does not play any role in this theoretical model. However, in real-world situations, we suspect this is not the case—it may be useful to consider various tree-traversal strategies depending on a letter-frequency, which is usually far from uniform.

### 4.4 Analysis of supersets

Our goal is to estimate an average time complexity for method getAllSubsets(*S*, *X*), and existsSuperset(*S*, *X*). Similarly as with subset-queries, since the length of output for getAllSubsets(*S*, *X*) is exponential in *α*_*k*_, we expect the obtained upper-bound to be exponential. For the function existsSuperset(*S*, *X*) we observe that, as long as *p* and *α*_*k*_ are not very small and fairly large, respectively, the expected number of visited nodes will not be large.

#### 4.4.1 Analysis of getAllSupersets(*S*, *X*)

When traversing the set-trie *S*, the algorithm visits the nodes in a depth-search manner and visits only the branches that do not miss any member of *X*. Let the random variable X represent the upper bound of the number of steps made when running the method *existsSuperset*(*S*, *X*). Since algorithm can only stop at *α*_*k*_-vertex, the full-tree of size $2^{\alpha_{k}}$ is a trivial upper-bound of number of getAllSupersets(*S*, *X*). Using the result from Proposition 7, we can obtain the upper-bound of the expected cardinality of the set-trie on an alphabet of size *α*_*k*_, which can be reduced to

$$\begin{array}{c} E(|X|)<\prod_{i=0}^{\alpha_{k}-1}[\frac{2+q^{2^{\sigma -i}}}{1+q^{2^{\sigma -i}}}]. \end{array}$$

The expression is clearly exponential in value of *α*_*k*_, i.e. the *rank* of the maximal element in X. Note that this bound is far from tight, as we do not consider the aspect of “branch pruning” in getAllSupersets and assume that the algorithm visits all the vertices in *S*[0, …, *α*_*k*_ − 1]. Nontheless, in the Experiment 3 we will observe that input sets with low-ranked members perform faster then these with high-ranked ones.

#### 4.4.2 Analysis of existsSuperset(*S*, *X*)

Using Bernoulli distribution, we now estimate the number of needed steps for existsSuperset(*S*, *X*). Although many of them will not be present, there is at most $2^{\alpha_{k}-k}$ possible positions of *α*_*k*_-node in *S*, and at the encounter of any *α*_*k*_-node, the algorithm stops immediately. If the algorithm failed to reach some potential *α*_*k*_-node in *S*, it needs to visit at most *α*_*k*_ additional nodes to come to the next one (or determine that it does not exist).

At any instance of *α*_*k*_-vertex, the algorithm stops with probability $p^{\prime }=(1-(1-p{)}^{2^{\sigma -\alpha_{k}}})$ or continues otherwise. If the algorithm does not stop after trying to visit all possible instances of *α*_*k*_-vertices, it returns False with probability $(1-p^{\prime }{)}^{2^{\alpha_{k}-k}}=(1-p{)}^{2^{\sigma -k}}$ after making at most $2^{\alpha_{k}-k}\cdot k$ steps. Let X be a random variable with the following distribution:

$$\begin{array}{c} P[X=ki]=\{\begin{array}{cc} p^{\prime }\cdot (1-p^{\prime }{)}^{i-1} & \;\text{if}\;1\leq i<2^{\alpha_{k}-k};\\ (1-p^{\prime }{)}^{2^{\alpha_{k}-k}-1} & \;\text{if}\;i=2^{\alpha_{k}-k}. \end{array} \end{array}$$

It is easy to check that this is a proper probability space that sums to 1. Indeed,

$$\begin{array}{cc} & p^{\prime }\cdot \sum_{i=0}^{2^{\alpha_{k}-k}-1}(1-p^{\prime }{)}^{i}+(1-p{)}^{2^{\alpha_{k}-k}}\\ = & p^{\prime }\cdot \frac{1-(1-p^{\prime }{)}^{2^{\alpha_{k}-k}}}{1-(1-p^{\prime })}+(1-p^{\prime }{)}^{2^{\alpha_{k}-k}}=1. \end{array}$$

**Theorem 13** Let A be the expected number of visited nodes in the algorithm existsSuperset(*S*, *X*), where *S* is a set-trie that corresponds to *W* ⊆ 2^Σ^, and X∈(Σk). Then A≤X. In particular,

$$\begin{array}{c} A<\alpha_{k}2^{\alpha_{k}-k}. \end{array}$$

**Proof 14** *Let* $w\prime =2^{\alpha_{k}-k}$, *let* $p^{\prime },X$ *be as defined above, and let q*′ = 1 − *p*′. *Again observe, that*

$$\begin{array}{c} \int \sum_{i=0}^{w_{X}}i\cdot q^{\prime i-1}dq^{\prime }=\frac{1-q^{\prime w_{X}+1}}{1-q^{\prime }}+C. \end{array}$$

*The calculations of the expectation of random variable* $X^{\prime }$ *below are similar than in previous section*:

$$\begin{array}{ccc} E(X^{\prime }) & = & \sum_{i}i\cdot P[X^{\prime }=i]\\ & = & \alpha_{k}\cdot w^{\prime }(1-p^{\prime }{)}^{w^{\prime }}+p^{\prime }\alpha_{k}\cdot \sum_{i=0}^{w^{\prime }}i\cdot (1-p^{\prime }{)}^{i-1}\\ & = & \alpha_{k}\cdot w^{\prime }(1-p^{\prime }{)}^{w^{\prime }}+\\ & & p^{\prime }\alpha_{k}\cdot \frac{\partial }{\partial (1-p^{\prime })}(\frac{1-(1-p^{\prime }{)}^{w^{\prime }+1}}{1-(1-p^{\prime })}+C)\\ & = & \frac{\alpha_{k}}{p^{\prime }}(1-(1-p^{\prime }{)}^{w^{\prime }}). \end{array}$$

**Fig 3:**
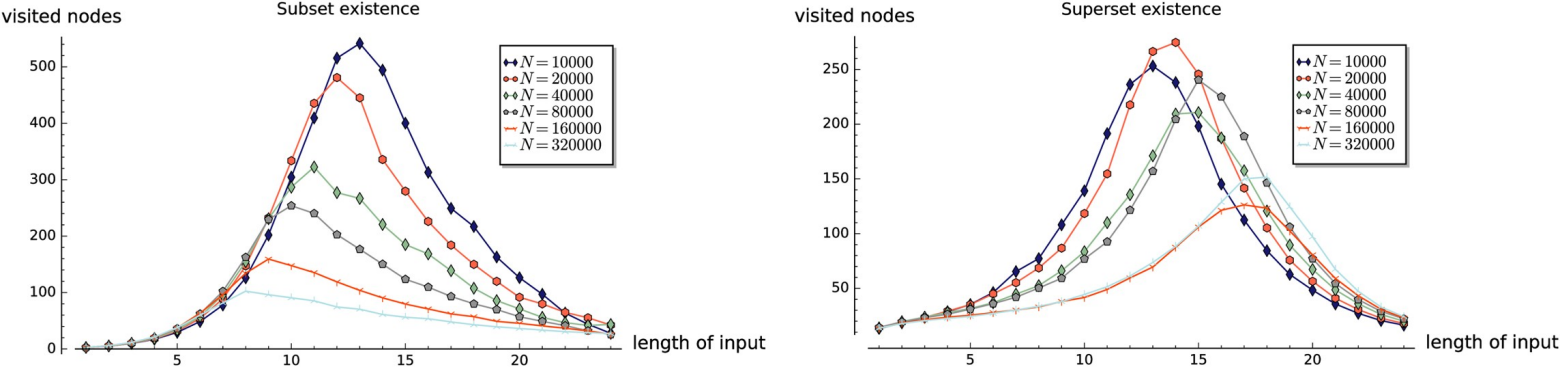
Experiment 1: Performance of existsSubset (*S*,*X*), and existsSuperset (*S*,*X*).

Again, notice that $\left(1-(1-p^{\prime }{)}^{w^{\prime }}\right)$ clearly lies in an [0, 1]. The obtained bound is useful whenever *p*′ is not very small, but the reader should notice that, while both *p* and *p*′ are members of [0, 1], it may often be the case that *p*′ ≫ *p*. For more intuition on the correlation between *p*, *p*′ and $\frac{\alpha_{k}}{p^{\prime }}$, and for easier correlation with Experiment 3, (see Fig 3), see some sample values on Table 1.

**Table 1:** The correlation between *p*, *p*′ and $\frac{\alpha_{k}}{p^{\prime }}$, with Σ = {0, …, 24} and various values of *p* and *α*_*k*_.

*α*_*k*_	12	6	17	24	5	14	24
*p*	10^−4^	10^−6^	0.03	0.08	0.0015	0.0015	0.0015
*p*′	0.559	0.408	0.999	0.154	1.000	0.954	0.003
$\frac{\alpha_{k}}{p^{\prime }}$	21	15	17	156	5	15	8006

One may observe that, as long as *p* and *α*_*k*_ are not very small and fairly large, respectively, the expected number of visited nodes will not be large. If, however, that would be the case, the results may not be as promising. The behavior of the time efficiency of the algorithm with various values of *p* can be observed in Experiment 1, while the relation with the value *α*_*k*_ is discussed in Experiment 3.

## 5 Empirical evaluation

This section presents the empirical study of the data structure set-trie. The performance of the set-trie is evaluated in a series of six experiments performed on the artificially generated and real-world data. The aim of the experiments is two-fold.

The first three experiments are designed to provide insight into the time complexity of the presented methods. The performance of set-trie is in these experiments measured by the number of nodes visited by the particular operation that we observe. As we measure the actual access frequencies (instead of measuring the absolute running time), this removes an unnecessary noise and enables a more precise insight into how our algorithms behave under different circumstances. The parameters and distributions for the random generation of our data aim to reflect the asymptotic time complexity of our data-structure in a natural way. The first three experiments are discussed in Section 5.1.

The last three experiments provide a real-world performance comparison between set-trie and the inverted index [2]. The inverted index is considered to be the most efficient data structure for storing sets of sets [4, 32]. The data used in the fourth experiment is generated with the data mining tool fdep. The last two experiments use the datasets msnbc and anonymous-msweb that contain the data about the access to the directory structures of two Web servers. The datasets were obtained from the UCI Machine Learning Repository [33]. These three experiments are presented in Section 5.2.

The data structure set-trie is implemented in the GNU C programming environment in the form of a library [28]. The set-trie was first implemented in the system for the induction of functional dependencies from relations fdep [26], also implemented in C, and, later, in the program for the induction of multi-valued dependencies implemented in Sictus Prolog [45].

### 5.1 Experiments on artificial data

In the experiments on artificially generated data, we are analyzing the influence of the selected parameters (e.g., the size of set-trie) on the performance of the data structure set-trie. The performance of set-trie is in these experiments measured by the number of nodes visited by the particular operation that we observe.

Let us present the basic terminology to be used for the description of the experiments. The alphabet used for the experiments is Σ = {1, 2, …, *σ*}, where *σ* is the size of the alphabet. The set trie *S* is constructed from a set of sets *W*. Each operation is tested by using the sets *X* from a test set of sets *T* of size *M*. The number of sets in a set-trie *S* is denoted by *N*. By the term *density* of the set-trie, we refer to the value of *N*/2^*σ*^.

Three experiments on the artificially generated data are presented in this section. In Section 5.1.1, we first present the procedure for the generation of sets that we use in Experiments 1-3. Experiment 1 is presented in Section 5.1.2. In this experiment, we study the influence of changing the size of a set of sets *W* and the corresponding set-trie *S* to the performance of set containment operations. The influence of the size of the alphabet Σ on the performance of the set containment operations is studied in Experiment 2 presented in Section 5.1.3. Finally, Experiment 3 presents the influence of the shape of the sets *X* ∈ *T*, i.e., the selection of the elements from Σ in the test set of sets *T*, on the performance of set-trie. This experiment is described in Section 5.1.4.

#### 5.1.1 The description of generating procedure and related notions

We construct the set-tries *S* from the collection of *N* subsets of Σ, where each subset is selected from P(Σ) uniformly at random, each by a given probability *p*. In other words, we traverse through all subsets of Σ and add each set to *S* with probability *p*.

The test set *T* of size *M* is generated in a similar way to the generation of the set-trie *S*. However, we construct *T* in a way that all lengths of the sets from *T* have approximately the same number of instances. Note that the parameters *p* and *M* have a direct influence on the cardinality of a given set-trie or test set, respectively.

#### 5.1.2 Experiment 1

In the first experiment we observe the influence of the size *N* of the set-trie *S* on the number of nodes visited in *S* by the operations existsSubset, existsSuperset, getAllSubsets, and getAllSupersets.

The size of the alphabet Σ is fixed to 25 so there can be at most 2^25^ = 33554432 sets constructed from Σ. The number of sets *N* in the set-tries *S* are: 10000, 20000, 40000, 80000, 160000 and 320000. Each operation is tested with the sets *X* from the test set *T* of the fixed size 50000.

The results of Experiment 1 for the operation existsSubset(*S*, *X*) are presented in Fig 3. When the size of *X* is small, the algorithm visits a few nodes to decide if a subset of *X* exists in *S*. The reason for this is in the small search space—only the elements of *X* need to be checked. Similarly, when the size of *X* is close to 25, only from 30 to 120 nodes are visited. In this case, most of the elements of Σ are included in *X*, and, therefore, some subset of *X* in *S* can be found quickly.

The maxima of functions for the operation existsSubset are around the sets of sizes 9-14. The subset of *X* in *S* may not be covered by the first path generated by the algorithm—the depth-first algorithm of existsSubset may backtrack a few times until the subset is found. The maxima for the set-tries *S* with the smaller number of sets, including from 10000 to 20000 sets, are around the sets of the size 13-14. The maximum is around the sets of size 9-10 for the larger set-tries storing from 160000 to 320000 sets. The shift of the peak to the left of the center is the consequence of the increased size of *S*, as it is presented in more detail in the sequel.

The algorithm of existsSuperset is, in a way, opposite to the algorithm of existsSubset. While the algorithm of existsSubset searches among the elements of *X*, the algorithm of existsSuperset must include all the elements of *X* and searches among the elements that are not in *X*. The set *X* in the algorithm of existsSuperset, therefore, acts as a constraint—the path in *S* that includes the superset of *X* must include all the elements from *X*.

The results of the operation existsSuperset(*S*,*X*) are presented in Fig 3. The number of visited nodes is significantly smaller for the operation existsSuperset in comparison to the number of visited nodes of the operation existsSubset. However, the number of visited nodes changes with regards to the number of sets in *S*. After some initial threshold in the number of sets in *S*, the performance of existsSubset becomes better than the performance of existsSuperset. This can be observed in Fig 3 where the performance of existsSuperset and existsSubsets flips while changing |*S*| from 80000 to 320000. The flip is obvious from the results of Experiment 2.

The peaks of the functions for existsSuperset in Fig 3 are, in comparison to the results of existsSubset, moved slightly to the right of the center point *σ*/2—the peaks move further to the right with the increasing size of *S*. The search for supersets is harder when the size of *X* is slightly larger than *σ*/2. The operation existsSuperset can find supersets more quickly when the size of *S* increases towards *σ*.

The figures of existsSubset and existsSuperset can be seen as mirrored across the line defined by *σ*/2. The explanation of this is the duality of the set operations subset and superset, as they define dual partial orderings of subsets of the alphabet Σ. This phenomenon is further studied in Experiment 3, where the duality of subset/superset operations is very obvious.

**Fig 4:**
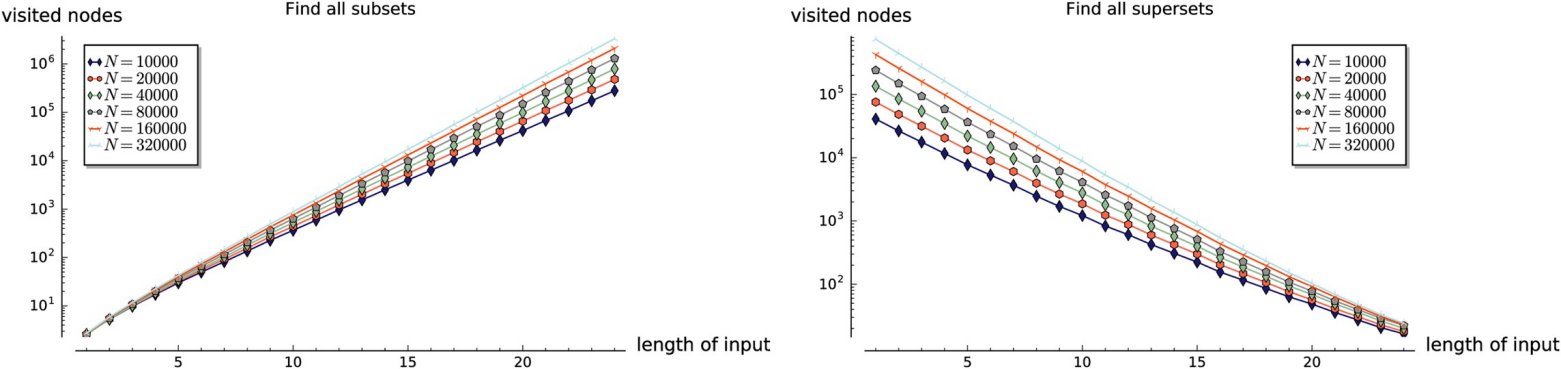
Experiment 1: Performance of getAllSubsets(*S*,*X*) and getAllSupersets(*S*,*X*).

Fig 4 shows the performance of operations getAllSubsets and getAllSupersets. The functions in Fig 4 are represented on a logarithmic scale. All functions are either growing or decaying linearly. This means that the number of visited nodes is either growing exponentially in the case of operation getAllSubsets, or decaying exponentially in the case of operation getAllSupersets. The behavior of the functions can be easily explained by seeing that the number of subsets in *S* grows exponentially with the size of *X*, and the number of supersets in *S* decays exponentially with the growing size of *X*.

#### 5.1.3 Experiment 2

In the second experiment, we study the influence of the size *σ* of the alphabet Σ on the performance of set-tries. The sizes *σ* used in experiments are 14, 17, 20, 23, and 26. For each particular case, the set of sets *W* is constructed, including 1.5% of all the subsets of Σ; for instance, in the case of *σ* = 24, we have approximately 250000 sets. The constructed sets of sets *W* are the inputs for the construction of the set-tries *S*. Furthermore, the test sets of sets *T* are constructed for each Σ to include 50000 sets.

First of all, let us note that because of the higher number of sets from *S*, the operation existsSubset visits fewer tree nodes than the operation existsSuperset. As we have described in the presentation of Experiment 1, the flip point, i.e., the point where the performances of subset/superset operations reverse, is, approximately, when there are 0.01 ⋅ 2^*σ*^ (1% of all possible subsets of Σ) sets in *S*. Since in Experiment 2, we have 1.5% of all possible sets in all cases, the results confirm the findings of Experiment 1.

**Fig 5:**
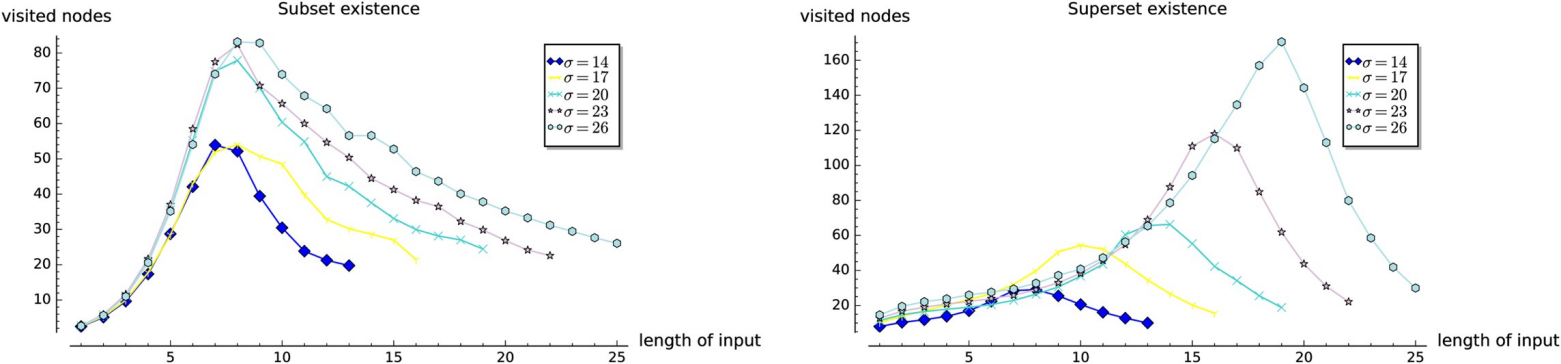
Experiment 2: Performance of existsSubset(*S*,*X*) and existsSuperset(*S*,*X*).

The results of Experiment 2 for the operation existsSubset(*S*, *X*) are presented in Fig 5. The maximal number of visited nodes for the alphabets of sizes 14, 17, 20, 23, and 26 are from 55 up to 85. The maxima of functions are around the elements with the indexes from 7 to 9, moving slightly to the right with the increasing size of the alphabet Σ. In the same way as in Fig 3 of Experiment 1, the peaks are actually moving slowly to the left of the central element with the increasing size of the alphabet Σ. However, the increase of the alphabet size causes the maximum to stay around the indexes from 7 to 9.

The results of the operation existsSuperset(*S*,*X*) are presented in Fig 5. The operation existsSuperset visits more tree nodes than the operation existsSubset, which is the consequence of the increase in the number of sets in *S*. The maxima of functions are always on the right-hand side of the middle elements of the alphabet Σ. They are moving further to the right with the increasing size of the alphabet Σ. The results are comparable to the results of Experiment 1, presented in Fig 3.

**Fig 6:**
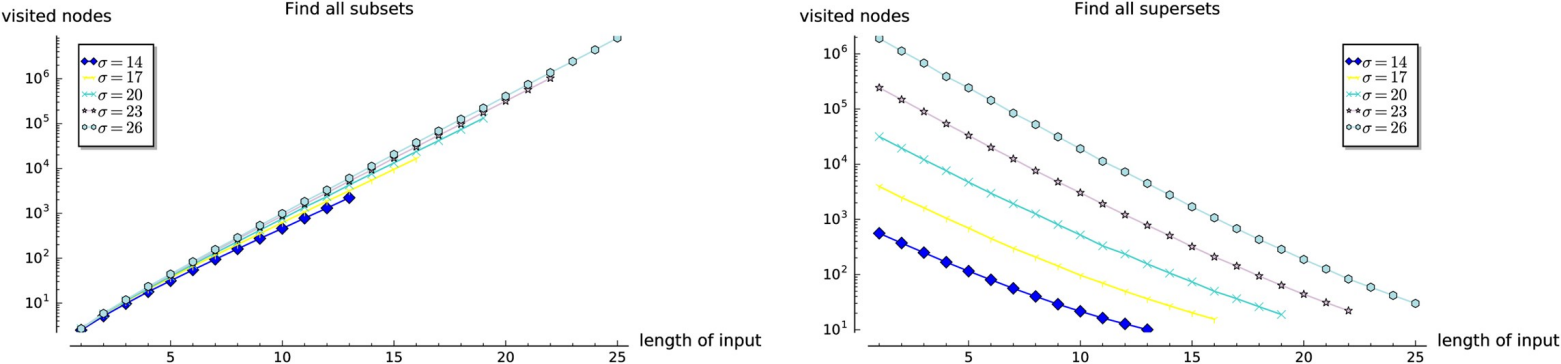
Experiment 2: Performance of getAllSubsets(*S*,*X*) and getAllSupersets(*S*,*X*).

Finally, Fig 6 shows the performance of operations getAllSubsets and getAllSupersets. Again, as in the case of Fig 4, the number of visited nodes of the operations getAllSubsets and getAllSupersets either increase or decrease linearly in the logarithmic scale, which means that the number of visited nodes either increases or decreases exponentially with the size of set *X*. This reflects the number of sets that are the results of the operations getAllSubsets and getAllSupersets.

#### 5.1.4 Experiment 3

This experiment is designed to provide more details about the behavior of operations when the input sets *X* include the elements from different ranges of the alphabet Σ. We show through the experiment that the operation existsSubset works faster if *X* predominately includes the elements with higher indexes and works slower in the case *X* includes the elements with lower indexes. The operation existsSuperset behaves oppositely: operation visits only a small number of nodes if *X* predominately includes the elements with lower indexes, and it visits a larger number of nodes if *X* includes the elements with the higher indexes. The experiment clearly shows the duality of operations existsSubset and existsSuperset.

Let us now present the experiment in more detail. The settings of the experiment are as follows. The size of the alphabet Σ is 25. We use 4 test sets *T* as the input of set containment operations. The test sets are named first, last, middle, and spread. Each of the test sets includes exactly 25 sets.

Let *k* in this paragraph stand for an index from [0, 24]. The set first includes the sets that for all *k* contain the indexes from the interval [0, *k*]. The set last includes 25 sets that contain the indexes from the interval [*k*, 24]. The set middle includes the sets that contain the elements around the center index 12. Each of them contains the elements from the range [(12 − ⌈*k*/2⌉), (12 + ⌊*k*/2⌋)]. Finally, the set spread includes 25 sets, one for each of the lengths *k*. These sets include the indexes that are spread as much as possible in the given range [0, 24]. The examples of the sets of the size 7, for each of the set of sets presented above, are as follows.

$$\begin{array}{c} \text{first}:\{0,1,2,3,4,5,6\}\\ \text{last}:\{18,19,20,21,22,23,24\}\\ \text{middle}:\{9,10,11,12,13,14,15\}\\ \text{spread}:\{0,4,8,12,16,20,24\} \end{array}$$

**Fig 7:**
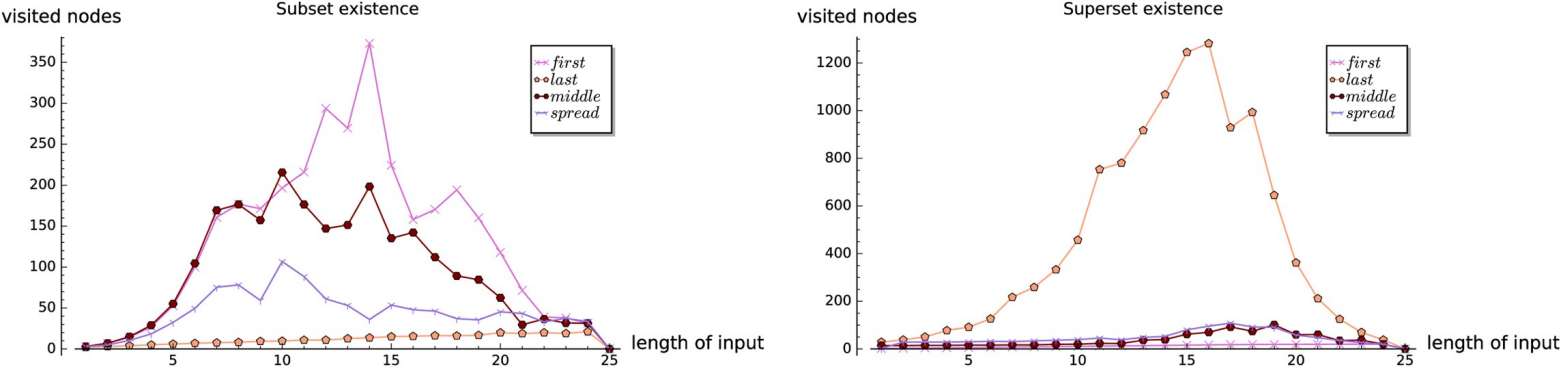
Experiment 3: Performance of existsSubset(*S*,*X*) and existsSuperset(*S*,*X*).

**Fig 8:**
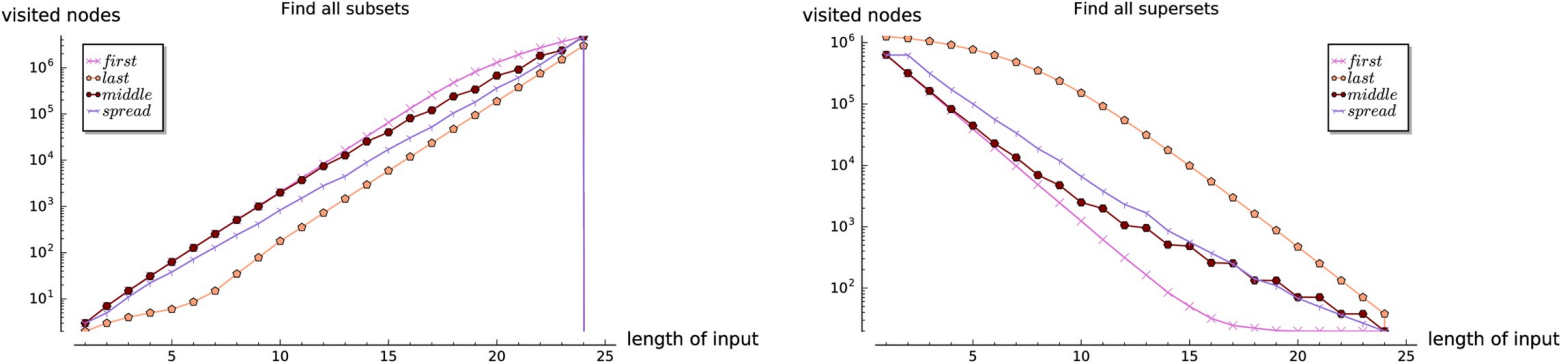
Experiment 3: Performance of getAllSubsets(*S*,*X*) and getAllSupersets(*S*,*X*).

The sets first, last, middle, and spread are tested on 20 different set-tries. The set-tries were generated by using the uniform distribution of the set elements as presented in Section 5.1.1. Each set-trie stores 1.5% of all possible subsets of [0, 24], i.e., approximately 500000 sets. The results for the set containment operations applied to the parameter sets first, last, middle, and spread, are presented as the four functions drawn in Figs 7 and 8. Note that each function value represent an average of 20 measurements.

Fig 7 shows the performance of operation existsSubset. The maximal number of visited nodes for the input set of sets first is for the sets of sizes from 12 to 16, where the number of visited nodes is from 250 to 370. This is more than it can be expected from the results presented in Fig 3 of Experiment 1. The reason for this is in the shape of the sets from first that causes the depth-first search algorithm of existsSubset to search on the left-hand side of set-trie where the largest number of sets is stored. The sets *X* from first do not include the elements with higher indexes, so in many cases, *X* does not cover the complete set of *S* with its search path. Therefore, the algorithm of existsSubset may search the middle area from the leftmost edge towards the center of the set-trie to find a subset of *X*. After the size of *X* is bigger than 16, the algorithm finds a subset much easier in the left-deep part of set-trie since *X*, in this case, includes more elements.

The results for the input set of sets last can be explained in the following way. When the sizes of sets from last are from 1 to 15, the search of existsSubset is performed on the sub-trees from the right-hand side of the set-trie where the number of nodes in sub-trees fall exponentially with the increasing index of the first element. The algorithm of existsSubset needs to visit a smaller number of nodes in smaller sub-trees. After the size of *X* is larger than 15, it is much easier to find the subset of a larger set, as in the case of first.

The number of nodes visited by the algorithm of existsSubset for the input set of sets middle is actually in the middle between the results obtained for first and last. Since the test sets *X* include indexes centered on 12, the sub-trees searched by the algorithm are smaller than in the case of the set of sets first. Therefore, operation existsSubset visits fewer nodes when applied to middle than when applied to first. Finally, since the shape of the sets from spread are close to the random sets used in Experiment 1, the results for the input set of sets spread are very close to the results of Experiment 1.

Let us now present the results of the operation existsSuperset. The results are presented in Fig 7. Firstly, the results of the operation existsSuperset when applied to first and last are the opposite to the results of the operation existsSubset. The algorithm of operation existsSuperset visits more nodes when applied to last than when applied to first. On the contrary, the operation existsSubset visits more nodes when applied to first than when applied to last.

A large number of nodes visited by the operation existsSuperset applied to last is the consequence of the shape of the input sets *X*, and the nature of the depth-first search algorithm of existsSuperset. The input sets *X* include in all instances the elements with the highest indexes {*k*, …, 24}. To check such an input set, the algorithm has to climb to the branches of the tree. Moreover, in the case that superset is not found, it has to climb into the next branch, etc. Since the algorithm of existsSuperset starts searching with the path including index 0 and then extends the path in a depth-first manner, it necessarily searches first the deepest part of the tree, progressing towards lower branches. Note that the path can include any element from {0, …, *k* − 1}. The performance of operation existsSuperset improves very quickly when the indexes of the parameter set *X* are moved down towards 0.

The results of the performance of existsSuperset on the parameter sets middle, and spread are similar to the results of Experiment 1. Indeed, the shape of input sets from middle, and spread are quite similar to the randomly generated input sets that are used for Experiment 1.

The case of first is a degenerated case. The algorithm searches for the path that includes all indexes in *X*, but it may include some other indexes that are not in *X*. In the case that the indexes of the input set *X* are not far apart, the algorithm of existsSuperset narrows the search while trying to include all elements from *X*. Since the sets from first are of the form {0, 1, …, *k*}, there is no need for searching, but the algorithm directly descends into some node, if the superset exists, or cannot find some element from *X* if the superset does not exist.

The performance of operations getAllSubsets and getAllSupersets is presented in Fig 8. The number of visited nodes grows exponentially with increasing the size of test sets *X* for the operation getAllSubsets, and, with decreasing the size of test sets *X* for the operation getAllSupersets. However, the shapes of the functions are quite different to those in Fig 4 of Experiment 1—they reflect clearly the results of the operations existsSubset and existsSuperset shown in Fig 7. The functions obtained for the operation getAllSubsets are the inverse of the functions obtained for the operation getAllSupersets. The functions of the test sets first and last represent the upper and the lower border for the operation getAllSubsets, and, they represent the lower and the upper border for the operation getAllSupersets.

### 5.2 Experiments on real data

In the three experiments on the real-world data, we compare the performance of set-trie with the performance of the inverted index. The indexes are implemented as in-memory data structures. Both indexes are implemented in the GNU programming language environment. The set-trie is implemented using the GNU C [28] and the inverted index in the GNU C++ [52]. The implementation of the inverted index is presented in Section 5.2.1.

Data for Experiment 4 are obtained by using the data mining tool fdep [26]. We have stored the sets that represent the intermediate results in the process of mining functional dependencies from the relational data. The experiment simulates parts of the algorithm for the induction of the functional dependencies where sets are used to represent the hypotheses. The presentation of Experiment 4, including the detailed presentation of the data obtained from fdep is given in Section 5.2.2.

Experiments 5 and 6 both use the data created by sampling and processing the logs from the two different Webs sites, namely msnbc.com and microsoft.com. The sequences in the datasets correspond to page requests of users during some period of time. The set containment queries inquire about the users that visited a given subset or superset of Web pages. The data and the results of the experiments are presented in Section 5.2.3.

#### 5.2.1 In-memory inverted index for storing sets of sets

The inverted index is implemented in the programming language C++ using exclusively standard libraries [52]. The data structure consists of two parts: dictionary and postings. In our case, the dictionary is implemented with the unordered_map. This is an associative container that stores key-value pairs with unique keys. The implementation of this container is guaranteed to have the average complexity for search operation *O*(1). Each unique key in the dictionary is mapped to a list of postings, which is implemented as a sorted vector. A single posting is a container for a set. A set is represented as a binary, in particular, a bitset of fixed length, such that the *n*−th bit is equal to 1 if the set contains the element *n*.

The set containment operations existsSubset, existsSuperset, getAllSubsets and getAllSupersets are implemented as also proposed in [32]. Suppose that the parameter set is called *X*. Each of the four operations requires first to retrieve the sorted postings for the elements of *X*. The superset queries compute the intersection of postings obtained for the elements of *X*. The sets *Y* obtained in this way have to contain all elements of *X* but can also contain additional elements.

The subset queries are implemented by checking all sets *Y* that appear in one or more (possibly all) postings of *X* elements. The selected sets *Y* are the subsets of *X* only if the size of *Y* equals the number of elements from *X* that include *Y* among their postings, i.e., *Y* ⊆ *X*. Note that all elements of postings have to be processed in the case of retrieval queries (retrieving all results) and in the case of the existence queries. The lengths of postings are used as a simple heuristics that guide the algorithms for joining the postings by selecting at each point of decision the smallest postings first [1].

#### 5.2.2 Experiment 4

This experiment uses the real-world data generated by the data mining tool fdep [26] for the discovery of functional dependencies from relations. The datasets Hepatitis and Lymphography from UCI Machine Learning Repository [33] is used as the input to fdep. The generated set of sets are split into test sets and sets used to construct a set-trie. The performance of the data structure set-trie is compared to the performance of the inverted index. The time in nanoseconds spent for each of the set containment operations is measured for set-trie and the inverted index.

Let us now present the setup and the contents of Experiment 4. First, the main features of the data mining tool fdep are described. Second, we present the procedure that is used to generate the sets of sets taken as the input of Experiment 4. Finally, we present the experiment and discuss the results.

**Datamining tool** fdep. The data mining tool fdep computes the set of valid functional dependencies from the input relation by using a lattice of sets as the main framework of the algorithms. In the basic algorithm, fdep enumerates the functional dependencies from the most general one towards more specific functional dependencies. The validity of functional dependencies is tested against the complete relation. The more efficient algorithm first computes from the raw relation the set of *invalid* functional dependencies. We refer to this set as the *negative cover*. The set of valid dependencies is then computed by testing valid dependencies against the negative cover. The set of valid dependencies is referred to as the *positive cover*.

**Generation of the input sets of sets**. The data used as the input of Experiment 4 consists of the unfiltered negative and positive covers generated by fdep from the datasets Hepatitis and Lymphography. The unfiltered negative and positive covers are the sets of functional dependencies that include redundant functional dependencies. For example, a functional dependency *Y* → *A* from a positive cover is redundant if *X* → *A* is also in the positive cover and *X* ⊆ *Y*.

The generated negative *NC* and positive covers *PC* are further split into the disjunctive sets based on the right-hand side attributes of functional dependencies. For a given dataset (e.g., Lymphography) with *k* attributes, we construct *k* disjunctive subsets *PC*_*i*_ of the positive cover, and *k* disjunctive subsets *NC*_*i*_ of the negative cover, where *i* ∈ [1, *k*]. To model precisely the set containment operations that appear in fdep, we base the experiments presented in this section on the sets of attributes that represent the left-hand sides of the functional dependencies from the sets *PC*_*i*_ and *NC*_*i*_. We refer to the generated sets of sets as *nc*_*i*_ and *pc*_*i*_, respectively.

In Experiment 4, we use four datasets obtained from the positive and negative covers generated by fdep from the domains Limphography and Hepatitis. The four datasets are named lymph.pc, lymph.nc, hepat.pc, and hepat.nc. Each dataset includes *k* sets of sets *c*_*i*_ for *i* ∈ [1, *k*]. From each *c*_*i*_, we take 20% of sets and use them as a test set of sets. The rest is used to construct a set-trie in one test run. For each *c*_*i*_, we measure the time spent by four set containment operations of the set-trie and the inverted index. The presented graphs show the average results for the *k* sets of sets from a given dataset.

**Fig 9:**
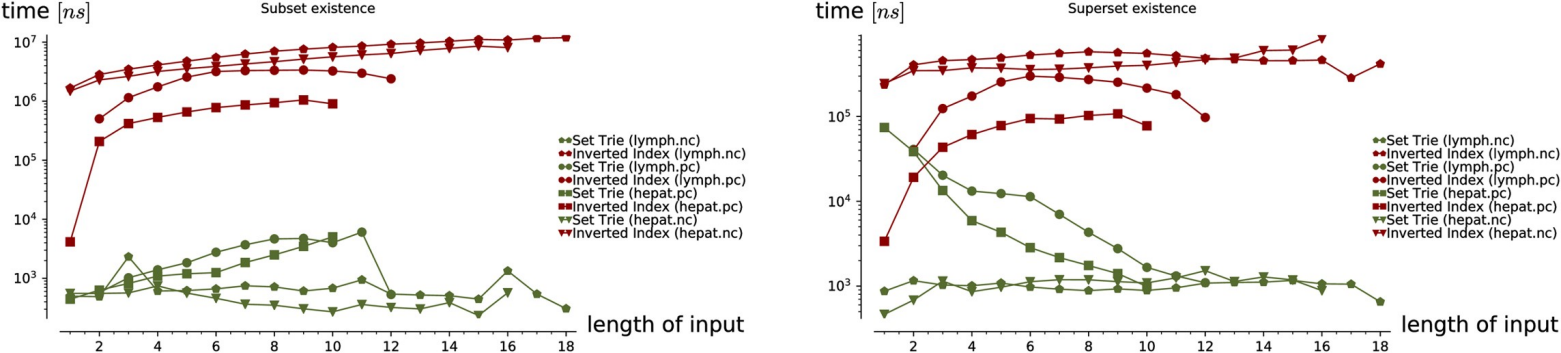
Experiment 4: Comparison of existsSubset(*S*,*X*) and existsSuperset(*S*,*X*).

**Fig 10:**
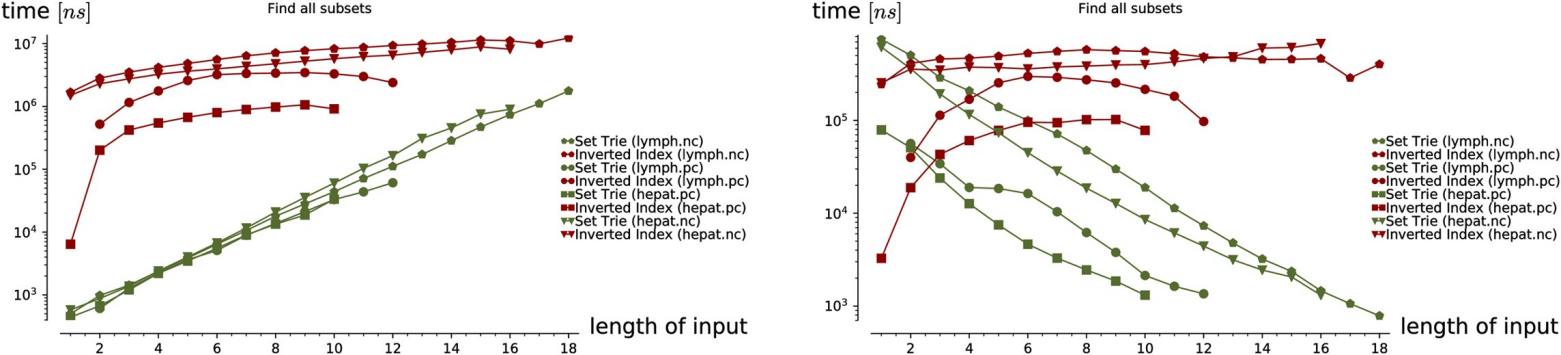
Experiment 4: Comparison of getAllSubsets(*S*,*X*) and getAllSupersets(*S*,*X*).

**Results of Experiment 4**. Experiment 4 presents the performance of the set-trie in comparison to the inverted index [2, 42]. The inverted index is considered to be the most efficient data structure for storing sets of sets [4, 32]. The results of the comparison are presented in Figs 9 and 10. In each figure, we show the results for all four set containment operations for both indexes, the set-trie and the inverted index.

The *x*-axis of the figures presents the sizes of the test sets used for measuring the time needed by the set containment operations to compute the result. The *y*-axis presents the time in nanoseconds (ns) needed on average to execute a given operation. The *y*-axis uses a logarithmic scale to be able to see the details about the faster methods. We can see from Figs 9 and 10 that set-trie outperforms inverted index by 1-3 orders of magnitude in most cases. Let us now give some comments about the behavior of the set-trie and the inverted index.

The data structure set-trie provides very fast existence queries, i.e., existsSubset and existsSuperset. From Fig 9 we can see that in most cases the existence queries need around 1 microsecond (1000 ns) to complete. On the other hand, the queries that return all subsets or supersets, i.e., getAllSubsets and getAllSupersets, need around 1 millisecond to complete, in the case of small test sets are the input to the operation getAllSupersets, and, in the case of large sets are the input to the operation getAllSubsets. In these two cases a large part of set-trie has to be searched to answer the query.

Figs 9 and 10 show that the inverted index needs approximately the same time for the existence queries and for the queries that retrieve the resulting sets. In cases of subset or superset existence operations, i.e., existsSubset and existsSuperset, the decision whether a superset exists in the inverted index can only be made after all posting lists are processed. In the same point, the operations getAllSubsets and getAllSupersets can start with the enumeration of the results. However, the computation time also depends on the size of the test set. If we search for the subsets or supersets of a small set, then the intersections of postings need less time to be computed.

#### 5.2.3 Experiments 5 and 6

In Experiments 5 and 6, we compare the set-trie with the inverted index on real data obtained from the log files of two Web servers. Both data structures are implemented in the GNU programming environment. The time measured in nanoseconds is used for comparing the performance of set containment operations. Besides comparing the running time, we also study the influence of the skewness on the performance of the data structures.

The datasets msnbc and msweb store the data about accessing the pages of two Web servers by random users. The msnbc dataset includes sequences of page accesses for 989818 users in a period of 24 hours. The Web pages of msnbc are classified into 17 categories that form the alphabet of our sets. The sequences are converted to sets that are used in Experiment 5. After the conversion to sets, there are only 10476 unique sets in the dataset. The original dataset includes a large number of one-element sets (600K). The number of sets for a given set size falls with the increase of the set size. In the original dataset, the average length of the sequences is 5.7, but the average size of the *all* sets is 1.7. The average size of unique sets is 5.9. The frequencies by which the elements of the alphabet are included in the sets in msnbc are relatively uniformly distributed.

The msweb dataset was created by sampling and processing the microsoft.com logs. The data represent access to 294 areas of the Web site by 37710 anonymous users. Each user is represented by a *set* (not a sequence!) of visited areas (vroots). The area numbers from msweb are mapped to the interval 1…294 that represents the alphabet of the set elements in Experiment 6. The sets include from 1 to 35 elements. There are 9994 sets of size 1. The number of sets for a given set size falls with the increase of the set size. The dataset msweb also includes repeating sets. There are 11233 unique sets in the dataset. In the original dataset, the average size of a set is 3. However, the average size of the unique sets is 5.2. The data in msweb is skewed. The frequencies of the appearance of the alphabet elements (Web site areas) in the sets are not uniformly distributed. For the details, see our statistics of the dataset given in [28].

The msnbc dataset is split randomly into 80% of sets that are used for the construction of the set-trie, and 20% of sets that are the test sets of Experiment 5. The msweb is provided in two parts: the data part consists of 32710 sets, and the test part includes 5000 sets. The data part is used for the construction of the set-trie, and the test part for the test sets of Experiment 6.

The set-trie is a data structure for storing sets of sets. Therefore, only the unique sets are stored in a set-trie. On the contrary, in the inverted index, each set has a unique identifier, and the duplicate sets are stored in the inverted index separately. The inverted index treats duplicate sets in the same way as any other set. Consequently, the inverted index has much more work because of the duplicates, while the set-trie relies on the unique sets when searching for subsets and supersets. To provide the conditions for a fair comparison, we use the unique sets from both datasets to construct the set-trie and the inverted index in Experiments 5 and 6.

**Fig 11:**
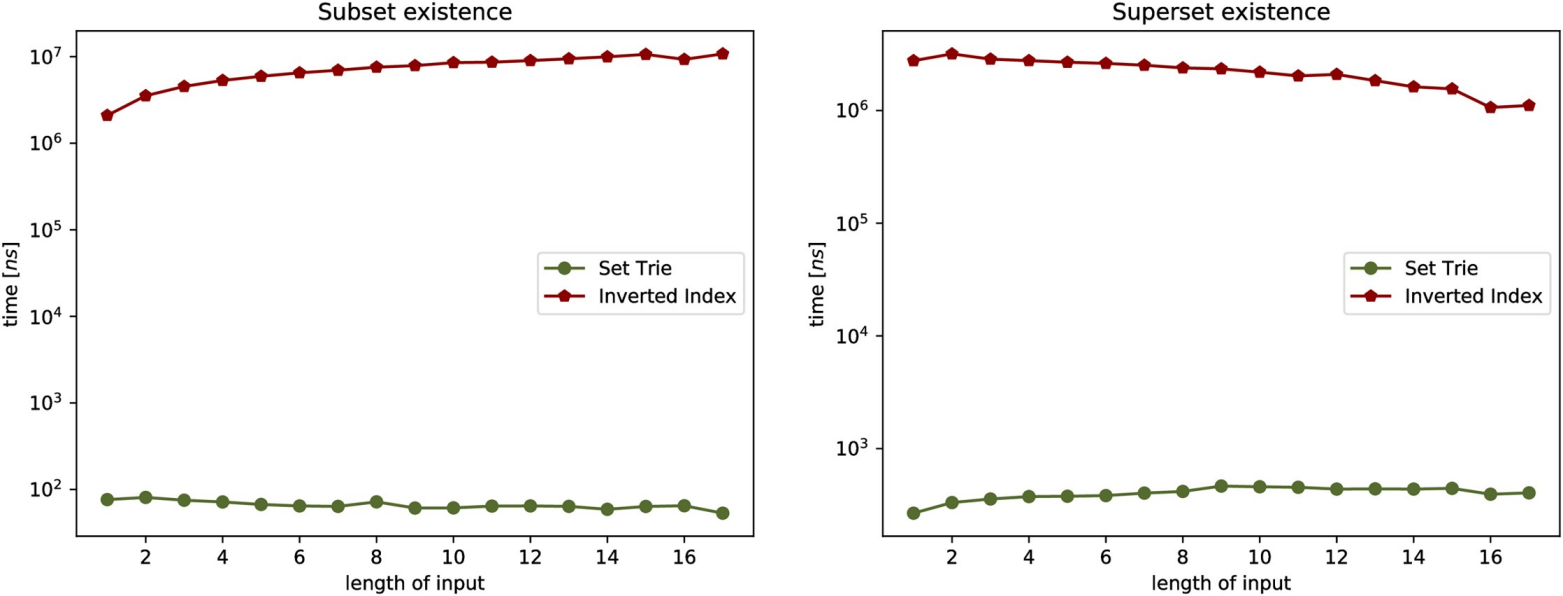
Experiment 5: Comparison of existsSubset(*S*,*X*) and existsSuperset(*S*,*X*).

**Fig 12:**
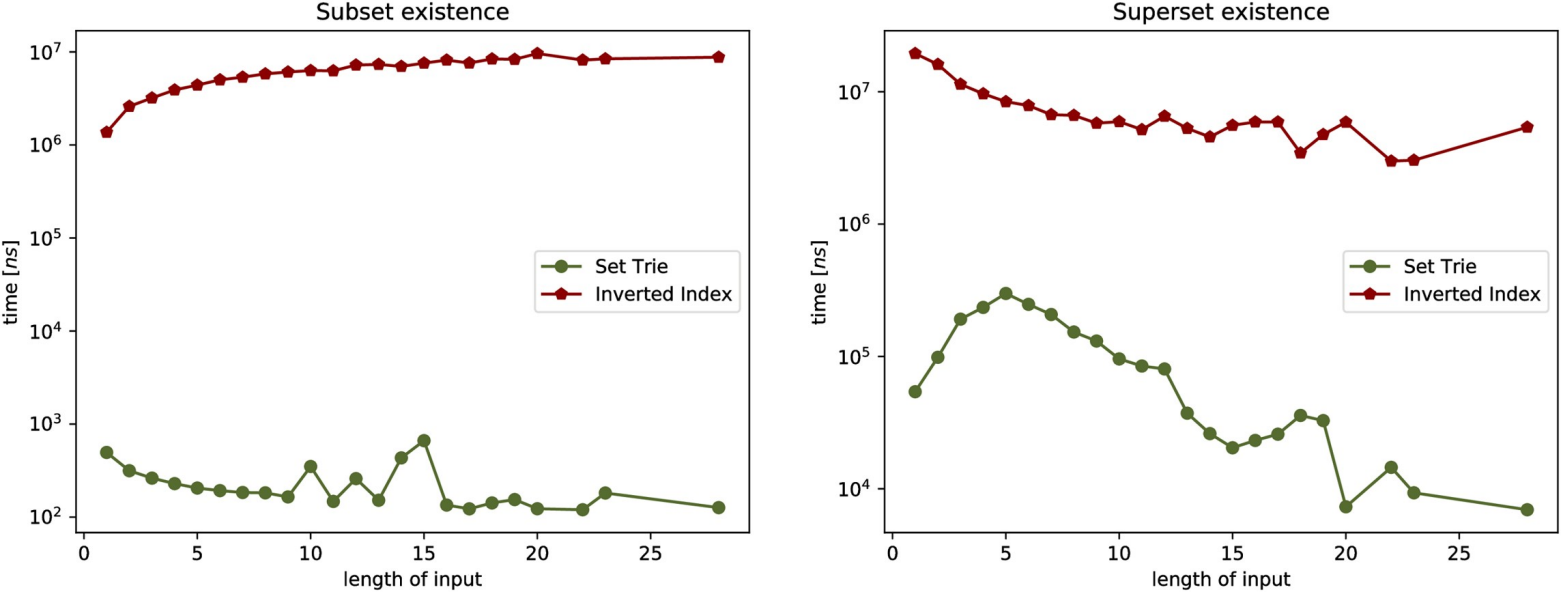
Experiment 6: Comparison of existsSubset(*S*,*X*) and existsSuperset(*S*,*X*).

Let us now present the results of Experiments 5 and 6. The performance of the operation existsSubset on the datasets msnbc and msweb are presented in Figs 11 and 12, respectively. In the set-trie, the operation needs around 100 nanoseconds to complete the task in Experiment 5, and from 100 to 1000 nanoseconds to finish in Experiment 6. The reason for this is that all possible single-element sets are stored in msnbc dataset, while the dataset msweb includes 190 single-element sets in the set-trie. For any parameter set *X*, the operation existsSubset immediately finds some single-element set included in *X*. In the inverted index, the operation existsSubset needs from 6 microseconds up to 10 milliseconds to complete the operation in Experiments 5 and 6.

The performance of the operation existsSuperset is presented in Figs 11 and 12. Let us first comment on the results of the set-trie. Fig 11 of Experiment 5 presents the time (in nanoseconds) spent by existsSuperset to find the first superset of a given parameter set *X* in the dataset msnbc. The operation needs a constant time of around 500 nanoseconds to return the result. The reason for this is a uniform distribution of the number of different sets for all sizes of sets. Thus, a superset very similar to the parameter set *X* can be found easily in the set-trie. Fig 12 of Experiment 6 presents the performance of existsSuperset on msweb dataset. The operation spends more time in the case that the parameter sets *X* are of smaller size. The reason for this is in the larger alphabet (294 elements) and in the correlation among the alphabet elements. The alphabet elements often appear in groups that are often visited together. For this reason, the operation spends more time searching for very specific supersets that may be dispersed in the set-trie. The operation existsSuperset spends less time for searching supersets in set-trie when the parameter sets are larger since there is a very small number of larger sets in the set-trie. In the inverted index, the operation existsSuperset needs from 1 to 5 microseconds for the msnbc domain in Experiment 5, and from 4 to 12 microseconds for the msweb domain in Experiment 6.

**Fig 13:**
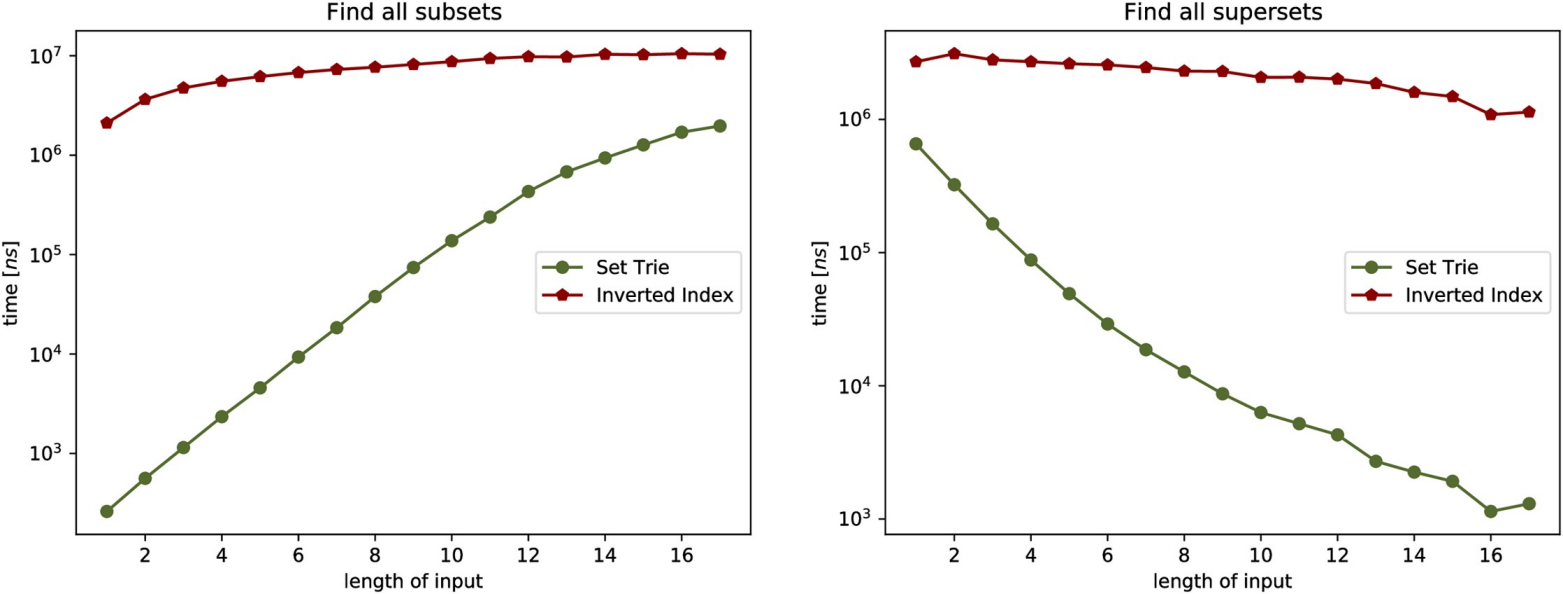
Experiment 5: Comparison of getAllSubsets(*S*,*X*) and getAllSupersets(*S*,*X*).

**Fig 14:**
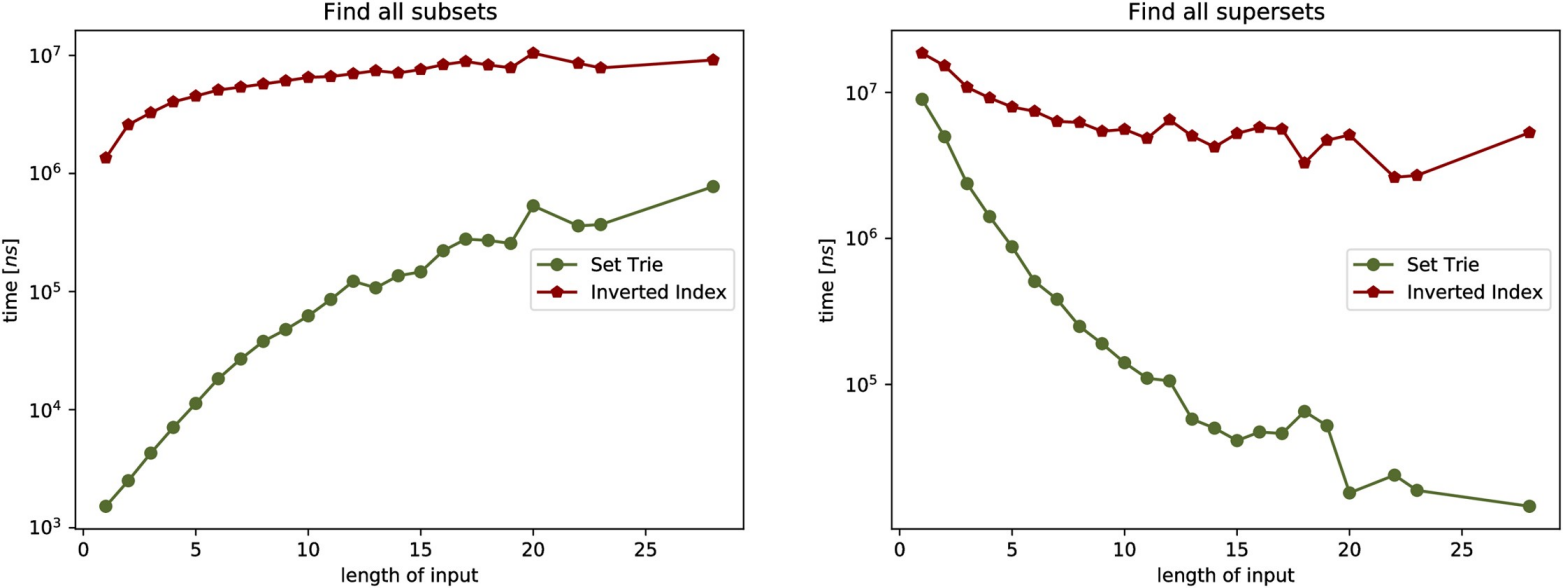
Experiment 6: Comparison of getAllSubsets(*S*,*X*) and getAllSupersets(*S*,*X*).

The performances of the operations getAllSubsets and getAllSupersets in Experiments 5 and 6 are presented in Figs 13 and 14, respectively. The results show that the set-trie outperforms the inverted index in both experiments by up to 3 orders of magnitude better running time.

Finally, let us give some comments on the influence of the skew on the performances of the set-trie and the inverted index. A dataset is skewed if the alphabet elements have significantly different frequencies of appearance in the dataset sets. The inverted index is sensible to the skewness [32, 46]. In the case that an alphabet element is a frequent member in sets, the inverted list for this element can grow very large. As a consequence, the set containment operations can perform significantly slower in the presence of skewness. On the contrary to the inverted index, the set-trie is robust to the skewness. The main reasons for this lie in the representation of sets, as well as in the procedures used for searching sets in a set-trie. The sets in the set-trie are represented as the sorted lists of set elements. The search involved in the set containment operations is based on this original representation of sets. The set containment operations search recursively among the neighboring sets stored in a trie. Therefore, the search does not depend on the frequency of the alphabet elements in sets.

## 6 Concluding remarks and future work

The paper considers a data structure set-trie for storing and querying sets. The efficient algorithms for the set containment operations are proposed. The algorithms are analyzed theoretically as well as empirically. The theoretical analysis gives some relevant upper bounds for the complexity of algorithms. In the empirical analysis, we thoroughly studied the search space of the proposed algorithms for set containment operations. The performance of set-trie is shown to be efficient enough for the storage and retrieval of sets in practical applications.

Experiment 1 and Experiment 2 indicate some of the very intuitive properties of the data structure set-trie. Experiment 1 shows that the number of the nodes visited by the set containment operations decreases roughly linearly with the exponential increase of the size of the set-trie *S*. It is easier to find a subset or a superset in the case there are more sets in the set-trie. Experiment 2 shows that, for the alphabet sizes 14-26, the number of visited nodes increases approximately linearly with the linear increase of the alphabet size.

The theoretical analysis, backed by the Experiment 3, shows that the efficiency of the presented algorithm is correlated with the choice of input characters. In particular, the ordering of the elements in the alphabet Σ may play an essential role in the process of algorithm design for the particular application. While the results of Experiment 3 may look very insightful, we are aware that the structure of each of four given inputs (first, last, middle, spread) is generated in a very artificial way. However, the results for the constructed test sets conveniently show how to order the alphabet. For instance, if we would like to speed up the operation existsSuperset, then the most frequent characters should be put at the end of the alphabet (see Fig 7). More studies would be needed to determine the optimal ordering of the alphabet in a given application environment.

The real-world datasets containing sets of low cardinality were used in three experiments where we compare the performance of the set-trie with the inverted index. Both data structures are implemented as in-memory indexes. Experiment 4 uses the sets generated while mining the functional dependencies from relations. Experiment 5 uses the msnbc dataset and Experiment 6 the msweb dataset. Both datasets contain sets of pages accessed by the visitors of a Web site. The cardinalities of the alphabets of sets from Experiments 1, 2, and 3 are 19, 17, and 294, respectively. The average cardinality of the sets generated using fdep is 9. The average cardinalities of unique sets from msnbc and msweb are approximately 6 and 5, respectively. We observe that the set-trie can store and query sets with alphabets containing up to several 100 and even 1000 elements. Furthermore, the set-trie can efficiently manage the datasets with very large alphabets only if the sets stored in the set-trie do not have a very large cardinality.

Finally, we noticed that the *set similarity queries* [20, 21, 25] can be implemented efficiently in the set-trie. For instance, an operation getAllSimilar(*S*, *X*, *N*) can be defined on the set-trie *S* to search for the sets similar to *X* where we can skip some elements from *X*, add some elements to *X*, but the number of changed elements of *X* must be less than *N*. The depth-first search strategy of the operation getAllSimilar can be seen as the combination of the search strategies of the operations getAllSupersets and getAllSubsets restricted by the number *N* of permissible changes of *X*. The search space of getAllSimilar is smaller than in the cases of getAllSupersets and getAllSubsets since it is controlled by the parameter *N*. We plan to perform the experiments that will study the complexity of getAllSimilar.
